# Unique Geothermal Chemistry Shapes Microbial Communities on Mt. Erebus, Antarctica

**DOI:** 10.3389/fmicb.2022.836943

**Published:** 2022-05-03

**Authors:** Stephen E. Noell, Mafalda S. Baptista, Emily Smith, Ian R. McDonald, Charles K. Lee, Matthew B. Stott, Jan P. Amend, S. Craig Cary

**Affiliations:** ^1^Te Aka Mātuatua-School of Science, Te Whare Wānanga o Waikato-University of Waikato, Hamilton, New Zealand; ^2^International Centre for Terrestrial Antarctic Research, University of Waikato, Hamilton, New Zealand; ^3^Interdisciplinary Centre of Marine and Environmental Research (CIIMAR/CIMAR), University of Porto, Matosinhos, Portugal; ^4^Faculty of Sciences, University of Porto, Porto, Portugal; ^5^School of Biological Sciences, University of Canterbury, Christchurch, New Zealand; ^6^Department of Earth Sciences, University of Southern California, Los Angeles, CA, United States; ^7^Department of Biological Sciences, University of Southern California, Los Angeles, CA, United States

**Keywords:** Mt. Erebus, Antarctica, geothermal, microbial community analysis, thermal gradient

## Abstract

Mt. Erebus, Antarctica, is the world’s southernmost active volcano and is unique in its isolation from other major active volcanic systems and its distinctive geothermal systems. Using 16S rRNA gene amplicon sequencing and physicochemical analyses, we compared samples collected at two contrasting high-temperature (50°C–65°C) sites on Mt. Erebus: Tramway Ridge, a weather-protected high biomass site, and Western Crater, an extremely exposed low biomass site. Samples were collected along three thermal gradients, one from Western Crater and two within Tramway Ridge, which allowed an examination of the heterogeneity present at Tramway Ridge. We found distinct soil compositions between the two sites, and to a lesser extent within Tramway Ridge, correlated with disparate microbial communities. Notably, pH, not temperature, showed the strongest correlation with these differences. The abundance profiles of several microbial groups were different between the two sites; class Nitrososphaeria amplicon sequence variants (ASVs) dominated the community profiles at Tramway Ridge, whereas Acidobacteriotal ASVs were only found at Western Crater. A co-occurrence network, paired with physicochemical analyses, allowed for finer scale analysis of parameters correlated with differential abundance profiles, with various parameters (total carbon, total nitrogen, soil moisture, soil conductivity, sulfur, phosphorous, and iron) showing significant correlations. ASVs assigned to Chloroflexi classes Ktedonobacteria and Chloroflexia were detected at both sites. Based on the known metabolic capabilities of previously studied members of these groups, we predict that chemolithotrophy is a common strategy in this system. These analyses highlight the importance of conducting broader-scale metagenomics and cultivation efforts at Mt. Erebus to better understand this unique environment.

## Introduction

Geothermal areas are dominated by microscopic life capable of thriving under extreme physicochemical conditions (Shu and Huang, 2021). The extreme selective pressures at geothermal sites drive the evolution of unique metabolic pathways and physiological adaptations in the resident microflora, challenging our perception of the limits of life (Fields, 2001; Belilla et al., 2021; Mueller et al., 2021). Terrestrial geothermal sites, distributed across the planet, are characterized by features such as hot springs, fumaroles, geysers, and localized hot soil sites (Inskeep et al., 2013; Mardanov et al., 2018; Power et al., 2018). Gradients in physicochemical parameters are commonly formed at the transition between isolated surface geothermal features and the surrounding non-geothermal environment. These gradients provide opportunities to examine how physicochemical parameters affect microbial community structures (Nakagawa and Fukui, 2002; Norris et al., 2002; Miller et al., 2009) and to explore microbial strategies for growth in these conditions (Takacs-Vesbach et al., 2013; Weltzer and Miller, 2013; Beam et al., 2014).

Studies of the impact of geothermal activity on microbial diversity in polar regions are limited, as these areas are often remote and difficult to access. Polar and subpolar geothermal sites include those in Iceland, Kamchatka, continental Antarctica, and sub-Antarctic islands such as Deception Island and the Sandwich Islands (Boyd et al., 1990; Marteinsson et al., 2001; Herbold et al., 2014b; Mardanov et al., 2018). The microbial diversity at the few confirmed geothermal sites in Antarctica, which include sites in Marie-Byrd Land and three volcanoes in Victoria Land, has been poorly studied (Broady et al., 1987; Bargagli et al., 2004; Soo et al., 2009; Herbold et al., 2014a). The three active volcanoes in Continental Antarctica are Mt. Rittman, which was discovered relatively recently and only harbors one geothermal site (Bargagli et al., 2004), Mt. Melbourne, a stratovolcano with geothermal features that include ice hummocks, ice towers, and hot soil spots (Broady et al., 1987), and Mt. Erebus on Ross Island.

**Figure 1:**
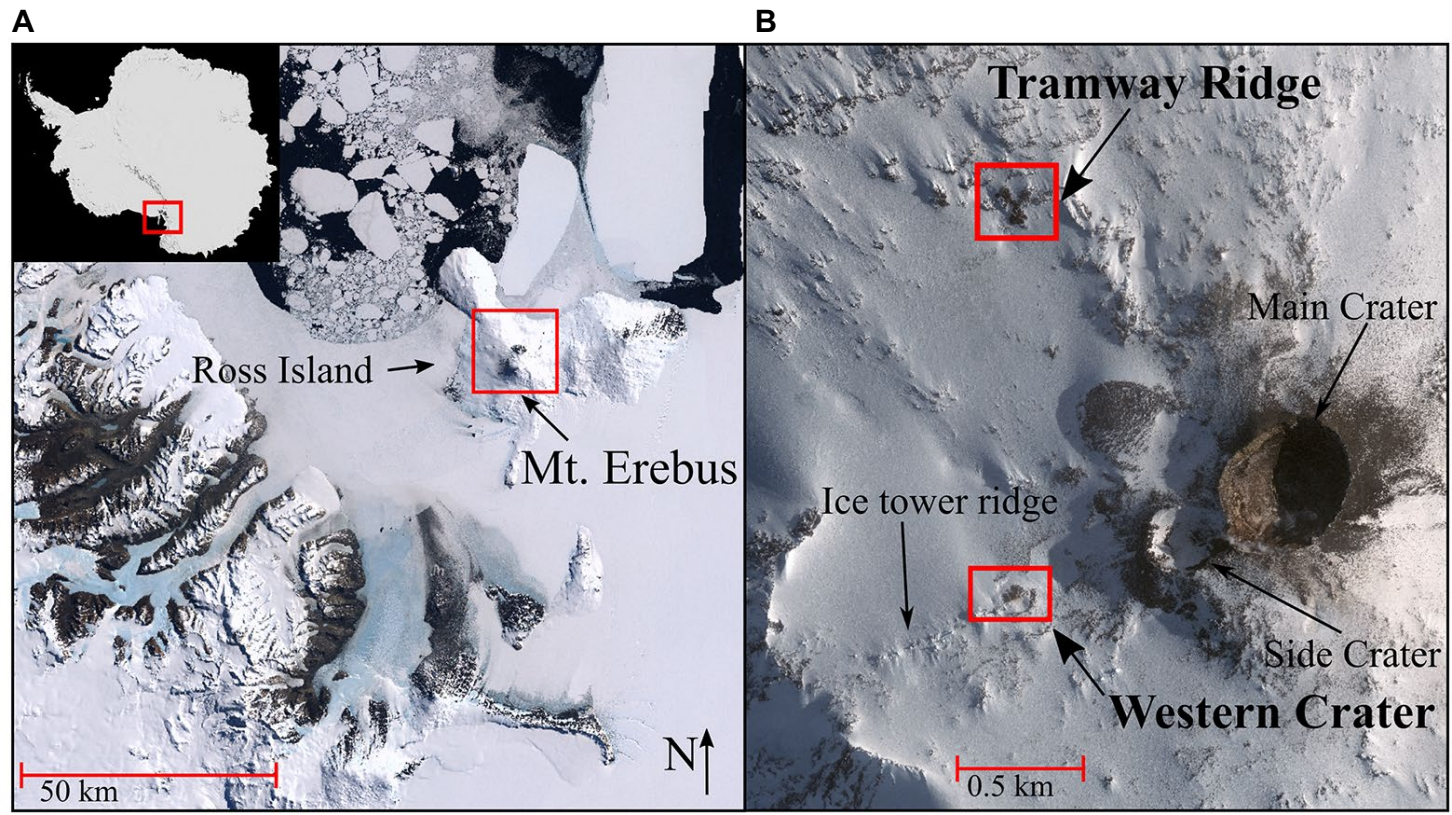
**(A)** Satellite image of Antarctica (top left inset, red rectangle indicates Victoria Land); main image shows Victoria Land and Ross Island, with Mt. Erebus (−77.53 and 167.17 DD) identified in the red rectangle. **(B)** The sampling sites on Mt. Erebus (red rectangles), Tramway Ridge (−77.519444 and 167.116389 DD) and Western Crater (−77.519167 and 167.187778 DD), and other main geothermal features on the Mt. Erebus summit. Satellite image in **(A)** is from the Antarctic Digital Database Map Viewer https://www.add.scar.org/, Open Source. Satellite image in **(B)** was purchased from DigitalGlobe Incorporated, Longmont CO United States (2019).

Mt. Erebus, at 3,794 m, is the second tallest volcano in Antarctica and the southernmost active volcano on the planet (Sims et al., 2021). Over the past decades, Mt. Erebus, classified as a phonolitic stratovolcano, and the many geothermal features on its summit have been extensively characterized (Kelly et al., 2008; Sims et al., 2021). Mt. Erebus is exceptional in its isolation from other large, active volcanic sites (Barker and Burrell, 1977), the presence of a persistent lava lake in the main crater that undergoes daily strombolian eruptions (Calkins et al., 2008; Kelly et al., 2008), its continuous gas production (Oppenheimer et al., 2011), and the high variation in gas composition across the summit (Ilanko et al., 2019). Many of the geothermal features are concentrated into five large geothermal fields: Main Crater, Side Crater, Western Crater, Tramway Ridge, and Ice Tower Ridge (Figure 1B). The geothermal features scattered across these fields include ice caves, ice chimneys, active steaming fumaroles, and large areas of ice-free hot soil. The persistently low air temperatures on Mt. Erebus result in the generation of steep temperature gradients moving away from individual geothermal features, with temperatures dropping over 60°C within only 10 cm (Soo et al., 2009). Probably the best-studied geothermal site on Mt. Erebus is Tramway Ridge, which lies at the terminus of an ancient lava flow and is relatively protected from the wind due to surrounding topography. Here, active fumaroles can reach 65°C, while the adjacent, slightly cooler soil supports well-established cyanobacterial mats and thick moss beds (Broady, 1984; Skotnicki et al., 2001).

Microbiological investigations at Mt. Erebus started with the isolation of novel strains of *Bacillus*, *Micrococcus*, and *Clostridium* (Ugolini and Starkey, 1966; Hudson et al., 1988, 1989; Hudson and Daniel, 1988; Nicolaus et al., 2001), resulting in the identification of a novel proteinase that is now commercially available (Coolbear et al., 1992). Culture-independent studies have been confined to Tramway Ridge (Soo et al., 2009; Herbold et al., 2014a; Vickers et al., 2016) and ice caves (Tebo et al., 2015). The Tramway Ridge studies found an abundance of novel and possibly endemic microorganisms, with pH and temperature being correlated with the microbial community structure and composition around individual fumaroles and increasingly novel microbial taxa with depth in the soil (Soo et al., 2009; Herbold et al., 2014a).

We undertook a comparative analysis of the physicochemical parameters and soil microbial communities across thermal gradients between two geochemically disparate sites on Mt. Erebus, Tramway Ridge, and Western Crater. These two sites were chosen to allow a comparison of a relatively sheltered site with high biomass, Tramway Ridge (Soo et al., 2009), and a more exposed site, Western Crater, which lacks active fumaroles and visible phototrophic mats (Panter and Winter, 2008). Two thermal gradients within Tramway Ridge were also analyzed to examine within-site heterogeneity at this higher productivity site. These three transects were examined through 16S rRNA gene amplicon sequencing and physicochemical analyses to assess the impact of temperature and geochemistry on microbial community composition and structure. We hypothesized that the distinct soil physicochemistry would correlate with distinct microbial communities between the two sites and within Tramway Ridge, supported by distinct metabolic strategies.

## Materials and Methods

### Site Description and Soil Collection

Soil samples were collected from two sites on Mt. Erebus, Antarctica, in November 2019. These included samples along two transects on Tramway Ridge (TR), 10 m apart (−77.519444 and 167.116389 DD), and one along a transect in Western Crater (WC; −77.519167 and 167.187778 DD; Figures 1A,B). Tramway Ridge is an Antarctic specially protected area (ASPA) at an elevation of approximately 3,450 m on the northwest slope of Mt. Erebus. It is an 80 m^2^ area located at the terminus of a phonolite lava flow with many active fumaroles that support extensive moss beds and cyanobacterial mats (Broady, 1984; Skotnicki et al., 2001). Western Crater is a smaller site with less fumarole activity (Wardell et al., 2003), located 1 km from Tramway on the southwestern side of Mt. Erebus at an elevation of 3,550 m, and with no noticeable moss or established cyanobacterial mats. Soil temperatures were measured *in situ* at both sites with a Checktemp 1C electronic temperature sensor (Hanna Instruments, Rhode Island, United States) to identify the greatest temperature soil (50°C–65°C) and establish a temperature gradient at 5°C–20°C intervals away from the hot spot (Supplementary Table 1). Samples were collected at these temperature intervals by discarding the top centimeter of soil and collecting the next 2 cm of soil using a sterile spatula. Samples were placed into sterile 50 ml tubes and frozen for transport back to the University of Waikato (Hamilton, New Zealand) for analysis.

### Geochemistry

Soil samples were characterized using a variety of geochemical analyses, conducted at the University of Waikato Analytical facilities. Elemental composition (B, Na, Mg, P, K, Ca, V, Cr, Fe, Mn, Co, Ni, Cu, Zn, Ga, As, Se, Sr., Ag, Cd, Ba, Tl, Pb, and U) was measured with an Agilent 89,000 ICP-MS (Agilent Technologies, Santa Clara, California, United States). Each sample (1 g) was ground using a Mixer Mill MM 400 (Retsch, Haan, Germany) at a frequency of 27.5 Hz for 1 min and 50 s, acid digested with HCl and HNO_3_ at 80°C for 30 min, diluted to 100 ml in MilliQ water, filtered with a 0.45 μM filter to remove soil particles, and diluted to an acid: water concentration of 1:5. A 10 ml aliquot from each sample was acidified with 200 μl of HNO_3_ before ICP-MS analysis.

pH and electroconductivity (EC) were measured by adding MilliQ water to soil samples at a 1:2.5 soil:water ratio. pH was measured using a HI2213 Basic pH/ORP/°C Meter /with 3-Point Calibration (Hanna Instruments, Rhode Island, United States). EC was measured using a thermo scientific orion 4-Star Benchtop pH/Conductivity Meter (Thermo Fisher Scientific). For gravimetric water content (GWC) measurements, 3 g of soil samples was dried at 105°C until the sample weight remained unchanged. GWC was calculated as the percentage of weight loss during drying (Herbold et al., 2014a). For total carbon and nitrogen measurements, dried samples were ground as above and 100 mg of each sample was measured on a CHNOS elemental analyzer (Elementar, Langenselbold, Germany) in the Stable Isotope Facility at the University of Waikato.

### DNA Extraction, Amplification, and Sequencing

Genomic DNA was extracted from 0.5 to 0.9 g of each soil sample using a CTAB/bead beating method (Herbold et al., 2014a), adapted by extending the incubation time to an hour. Where DNA yield was low, multiple (2–3) extractions were carried out and the DNA subsequently pooled. Genomic DNA was quantified using a Qubit 2.0 Fluorometer (Thermo Fisher Scientific, Massachusetts, United States).

The 16S rRNA gene (V4-V5 region) was amplified by PCR using the 515F (Parada) and 926R (Quince) primers (Caporaso et al., 2018; Ul-Hasan et al., 2019), adapted as fusion primers for Ion Torrent sequencing by including a unique tag and IonXpress barcode to distinguish individual samples and enable Ion Torrent sequencing (Whiteley et al., 2012). This primer set exhibits very high coverage of archaea and bacteria and is one of the most widely used (Parada et al., 2016). The 20 μl reaction mixture included 0.24 mM dNTPs, 1.2 × PCR buffer, 6 mM MgCl_2_, 0.016 mg/ml BSA, 0.2 mM of each primer, 0.024 U Taq polymerase (Thermo Fisher Scientific, Massachusetts, United States), and 9 ng of genomic DNA, except for one sample (WC20) that contained 3 ng DNA due to low DNA yield. The reaction conditions were as: initial denaturation (94°C), 3 min; 30 cycles of 94°C for 45 s, 50°C for 1 min, and 72°C for 1.5 min; and final extension was 72°C for 10 min. All PCR reactions were run on an Applied Biosystems ProFlex PCR System (Thermo Fisher Scientific). PCR reactions were run in triplicate to account for possible PCR bias; quality was checked using electrophoresis gels. Following pooling of triplicate PCR products from each sample, 25 μl of each was treated with Invitrogen SequalPrep Normalization (Thermo Fisher Scientific) to purify, normalize the PCR product concentration, and remove DNA fragments smaller than 100 bp. The Ion Torrent amplicon library was constructed using 2 μl from each purified PCR product and quantified *via* Qubit 2.0 Fluorometer (Thermo Fisher Scientific). The library was prepared for sequencing using the Ion PGM™ Template IA 500 preparation kit and the Ion PGM™ Hi-Q™ View Sequencing kit (Thermo Fisher Scientific) and was then added to an Ion 318™ Chip Kit v2 BC (Thermo Fisher Scientific). Samples were sequenced on an Ion Torrent PGM (Thermo Fisher Scientific) at the University of Waikato DNA Sequencing Facility.

### Sequence Quality Control and Taxonomic Assignment

The raw 16S rRNA gene amplicon sequences from the Ion Torrent PGM were processed using the DADA2 pipeline according to a previously published workflow using default settings with additional parameters recommended for Ion Torrent (HOMOPOLYMER_GAP_PENALTY = –1, BAND_SIZE = 32; Callahan et al., 2016). Chimeras were removed with the removeBimeraDenovo function using the method “consensus.” Taxonomy was assigned with the function assignSpecies using the native implementation of the naive Bayesian classifier and the SILVA database version 138.^1^ Eukaryotic, mitochondrial, and chloroplast sequences were removed and an unrooted phylogenetic tree was built with the neighbor-joining method, maximizing the likelihood with a gamma model distribution, using the decipher (Wright, 2016) and phangorn (Schliep, 2011) packages in R.

After 16S rRNA gene sequence reads were filtered for quality, four samples (TR152, TR234, WC20, and WC40) showed lower read counts than the rest of the samples (3,000–8,000 reads compared to >15,000 reads; Supplementary Figure 1A). Despite this, the median read count of samples from each transect was similar (25,000–30,000; Supplementary Figure 1B). When the Unifrac distances between samples were plotted using Principal Coordinates Analysis (PCoA) for either all samples or the set without the four low-read samples, a very similar amount of variation was explained by both axes (36.9% vs. 35.3% for axis 1, with/without low-read samples, respectively; Supplementary Figures 1C,D). Finally, rarefaction curves of ASVs showed that the low-read samples reached a plateau, indicating that the sampling of microbial diversity in these low-read samples was adequate (Supplementary Figure 1E). For these reasons, combined with the limited number of these rare samples to work with, all biological samples were retained for further analyses.

### Statistical Analyses

All statistical analyses were conducted in R (R Core Team, 2020), with plots obtained with ggplot2 v3.3.5 (Wickham, 2009) and edited for esthetics in Inkscape^2^; data analysis was performed with tidyverse v1.3.1 (Wickham et al., 2019). A two-sided t-test was performed to test for pairwise differences in physicochemical factors between different sampling site locations. A Spearman’s correlation coefficient was performed to test for significant correlations between temperature and environmental parameters or elements at either of the sites or either of the Tramway Ridge transects. Microbial community structure was assessed with a principal coordinates analysis (PCoA) of center log-ratio transformed (CLR) sequence abundance on a Unifrac distance matrix using phyloseq v1.32.0 (McMurdie and Holmes, 2013). CLR transformation was performed using the microbiome package v1.12.0 (Lahti and Shetty, 2017). Statistical differences were evaluated with a permutational analysis of variance (PERMANOVA) after testing that samples did not differ significantly in their dispersion by an analysis of multivariate homogeneity (PERMDISP), both using the R package vegan v2.5–7 (Oksanen et al., 2020). Alpha diversity was also assessed with the R package vegan, using the Shannon index. Venn diagrams were plotted using the R package VennDiagram v1.16.20 (Chen and Boutros, 2011) to portray the unique ASVs in each transect. A Mantel test with 999 permutations was employed to correlate physicochemical factors with the microbial community structure, using the vegdist function in vegan. Factors identified as significant by this test (*p <* 0.05) were plotted using a distance-based Redundancy Analysis (dbRDA) in the capscale function from vegan with the CLR transformed sequence abundances used above.

### Random Forest Analysis

Random Forest modeling employing the R package randomforest v4.6–14 (Liaw and Wiener, 2002) based on 1,000 decision trees was used to predict the importance of each ASV for distinguishing between the two Tramway Ridge transects and between the Tramway Ridge and Western Crater sites. Before the Random Forest modeling, the filtered ASV set was trimmed to reduce noise, retaining ASVs that had >10 reads in >2 samples, which reduced the number of ASVs from 940 to 350. Read counts of remaining ASVs were CLR transformed as above. The ability of the random forest to classify ASVs based on site and transect was assessed with the package caret (v6.0.86) and exhibited an accuracy (area under the receiver operating characteristic curve) of 1 (95% confidence interval = 0.59–1, *value of p* = 0.02; Kuhn, 2017). To identify the key ASVs for distinguishing sites, ASVs identified by the Random Forest algorithm as having a mean decrease Gini value (MDG) above zero were plotted on a scree plot (Roguet et al., 2018). The breakpoint of this scree plot was found to be an MDG of 0.06; ASVs with an MDG above this value were retained for plotting.

### Network Analysis

Co-variation network analysis was performed to determine the co-variation of ASVs in our data set. The analysis was conducted in R with the 16S rRNA gene amplicon sequence dataset, trimmed to retain ASVs with >10 reads in >3 samples (217 ASVs) for ease of visualization. The network was calculated with the SPIEC-EASI function in the SpiecEasi package v1.1.1 (Kurtz et al., 2015) using the Meinshausen-Buhlmann Neighborhood Selection method with 50 repetitions. The mean CLR abundance of ASVs was used as the node size. The modularity of the network was calculated using igraph v1.2.6 (Csardi and Nepusz, 2006) with the undirected fast greedy modularity optimization algorithm. The network was plotted using the plot_network function of phyloseq. Due to the small number of overlapping ASVs between sample sites and the small sample set we are working with, our resulting network cannot distinguish co-occurrences that are the result of interacting microbes and those that are due to similar habitat preference (Berry and Widder, 2014).

## Results

### Soil Geochemistry Characterization

Tramway Ridge and Western Crater samples showed different physicochemistry. This was apparent from an initial Principal Coordinates Analysis (PCoA), where Western Crater samples clustered along both axes (Supplementary Figure 2A). This pattern was more pronounced when elemental chemistry data were removed from the analysis, which also increased the amount of variation explained by the primary dimension (61% without elemental chemistry vs. 40% with elemental chemistry; Supplementary Figure 2B).

**Figure 2:**
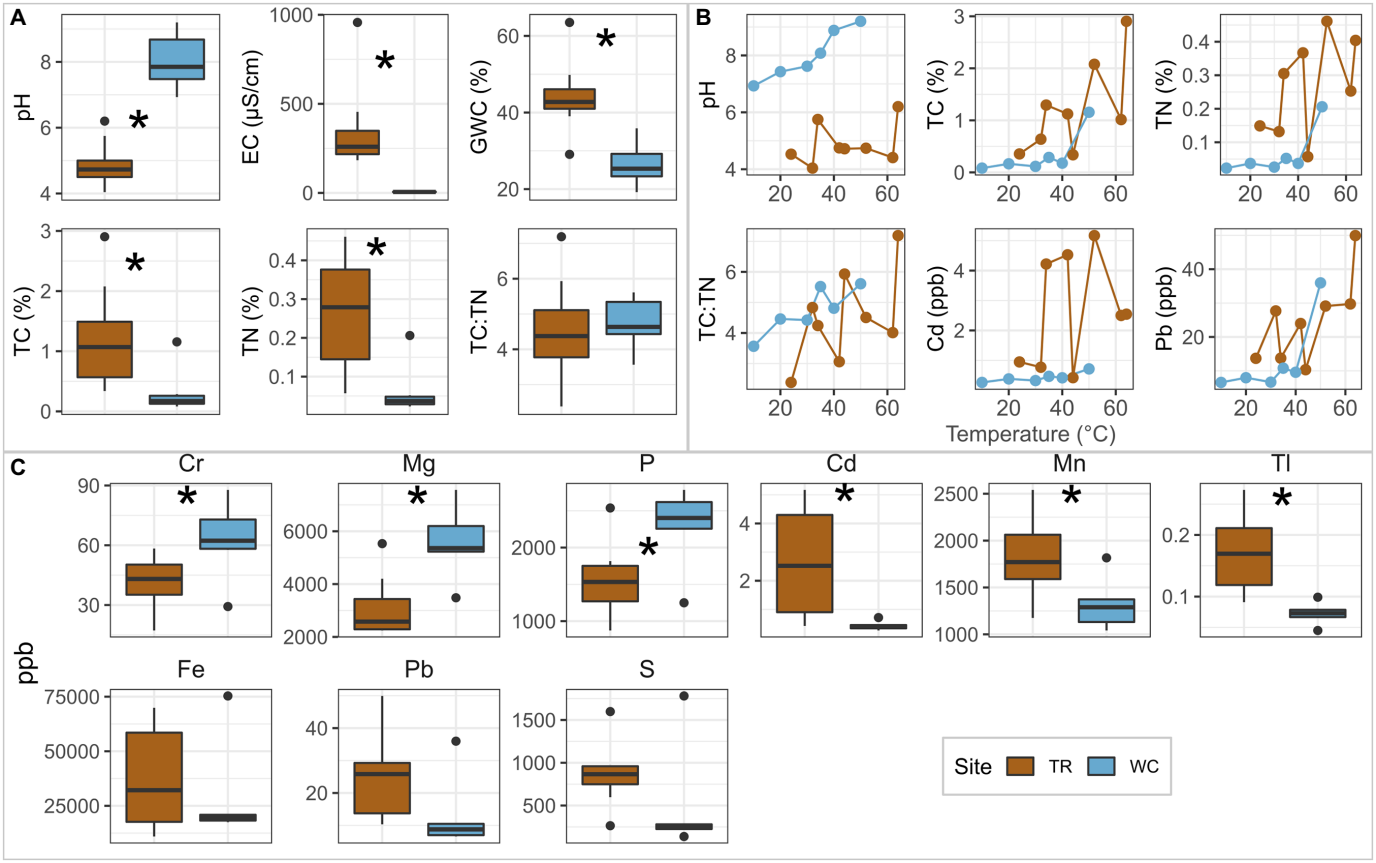
Comparison of soil physicochemistry between Tramway Ridge (TR) and Western Crater (WC). **(A,C)** Boxplots for **(A)** each environmental parameter and **(C)** elements of interest that were significantly different between the two sites, showed significant trends with temperature, or are potential substrates for microbial metabolism. **(B)** Line plots showing the variation with temperature of environmental parameters and elements that were significantly correlated (Spearman’s correlation coefficient, *p* < 0.05) with temperature at one of the two sites; all significant correlations were found at WC. Elements were grouped by their patterns of difference between sites. * indicates significant difference between samples from the Western Crater transect and both Tramway Ridge transects (two-sided *t*-test, *p* < 0.05). Ppb: concentration in parts per billion. GWC: gravitational water content. TC: total carbon; TN: total nitrogen.

The average values of many physicochemical parameters were significantly different between the two sites (two-sided *t*-test, *p <* 0.05; Figure 2, Supplementary Table 1; elements shown are significantly different between sites, had significant trends with temperature, or are potentially important from a microbial metabolism perspective; all measured elements are provided in Supplementary Table 2). The average pH was greater at Western Crater (8.02; ranged from 6.93 to 9.20) than Tramway Ridge (4.89; ranged from 4.04 to 6.20; *p =* 3.9E-5; Figure 2A). Western Crater had significantly lower average values than Tramway Ridge for gravimetric water content (GWC; *p =* 0.001), electroconductivity (EC; *p =* 0.006), total carbon (TC; *p =* 0.03), and total nitrogen (TN; *p =* 0.005; Figure 2A). The concentrations of multiple elements were significantly different between the two sites; several other elements, notably S, had higher average values at Tramway Ridge than Western Crater (864 ppb at Tramway Ridge vs. 487 ppb at Western Crater for S), although this difference was not significant (Figure 2C). Tramway Ridge also had increased Pb^206^/Pb^207^ ratios compared to Western Crater (Supplementary Figure 4B, Supplementary Table 2). Several parameters and elements had significant, positive correlations with temperature at Western Crater (Spearman’s correlation coefficient, *p <* 0.05); notably, pH showed an increase with temperature at Western Crater, but not Tramway Ridge (Figure 2B, Supplementary Figure 4B).

Within Tramway Ridge, the two individual transects sampled showed distinctive profiles despite many similarities in soil geochemistry (Supplementary Figure 4). Tramway Ridge transect 2 (TR2) had higher averages than transect 1 (TR1) for pH and EC (Supplementary Figure 4A), although these differences were not significant (*p =* 0.1 and 0.4 for pH and EC). Similarly, when comparing the same elements of interest as identified in the between sites comparison, TR2 had higher, albeit non-significant, average concentrations of several elements (Supplementary Figure 4C). TR1 had a much higher average Pb^208^/Pb^207^ ratio than TR2 (Supplementary Figure 4B, Supplementary Table 2).

### Effects of Geochemistry on Diversity

Tramway Ridge samples had greater DNA yield (0.02–3.3 μg DNA/g soil) than Western Crater (0.005–0.164 μg DNA/g soil; Supplementary Table 1). The alpha diversity (Shannon Index) ranged from 2.5 to 5.0 across all samples, with the largest species richness at Western Crater (Supplementary Figure 5A). A total of 940 ASVs, 25 archaeal and 915 bacterial, were identified across all samples, with the largest number of unique ASVs (496) at Western Crater (Supplementary Figure 5B). The two Tramway Ridge transects shared the most ASVs (106; Supplementary Figure 5B). Only 43 ASVs, comprising 5% of total ASVs, were found at all three transects (Supplementary Figure 5B). 6% of all reads could not be assigned to a phylum and 58% of reads were unassigned at the genus level, highlighting the large number of novel microbes inhabiting Mt. Erebus geothermal soils.

The microbial community composition at Western Crater was significantly different from the composition at both Tramway Ridge transects (*p <* 0.01), but no significant difference was found between the two Tramway Ridge transects (*p =* 0.15; pairwise PERMANOVA test). When biological samples were plotted using Unifrac distances with PCoA, the primary axis (explained 33.1% of variation) separated Western Crater samples from the Tramway Ridge samples (Supplementary Figure 2C), as in the environmental data (Supplementary Figure 2B), indicating these could be correlated. Again, no clustering was observed within the two Tramway Ridge transects (Supplementary Figure 2C).

**Figure 3:**
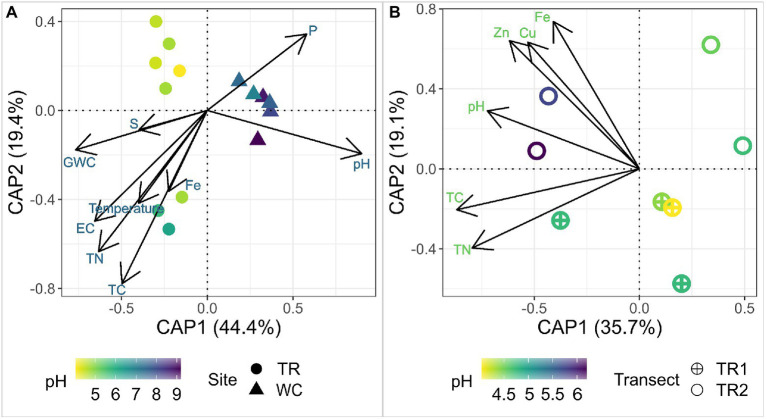
A distance-based Redundancy Analysis (dbRDA) of 16S rRNA gene ASV profiles, which were center log-ratio transformed and constrained by the physicochemical parameters identified as significantly (Mantel test, *p* < 0.05) affecting the microbial community composition either **(A)** between Western Crater and Tramway Ridge, or **(B)** within Tramway Ridge. Ordination was constructed using Unifrac distances among samples. The shape represents the site/transect of origin for the sample, color represents the pH of the sample as pH was identified as being most important for distinguishing samples between sites. The percentages on the axes correspond to the variance in biological samples explained by the combination of the selected environmental parameters. The arrow direction for each environmental factor represents the variance direction for that parameter. TR: Tramway Ridge site; WC: Western Crater site; TR1: Tramway Ridge transect 1; TR2: Tramway Ridge transect 2; GWC: gravitational water content; EC: electroconductivity; TC: total carbon; TN: total nitrogen.

To explore the impact of environmental factors on the microbial diversity at these sites, a distance-based Redundancy Analysis (dbRDA) was employed using physicochemical parameters and elements that were significantly correlated (Mantel test, *p <* 0.05) with the microbial communities (Figure 3A). The high pH and P of Western Crater samples were associated with community composition (Figure 3A). The community composition of Tramway Ridge samples was associated with multiple parameters, especially GWC, EC, TN, and temperature (Figure 3A). Within Tramway Ridge, pH, Fe, Cu, and Zn were associated with the microbial community composition at the highest pH samples (TR2-64, TR2-34, and TR1-52; Figure 3B). Thus, pH was found to be the most important determinant of community composition both between sites and within Tramway Ridge.

### Random Forest Analysis

**Figure 4:**
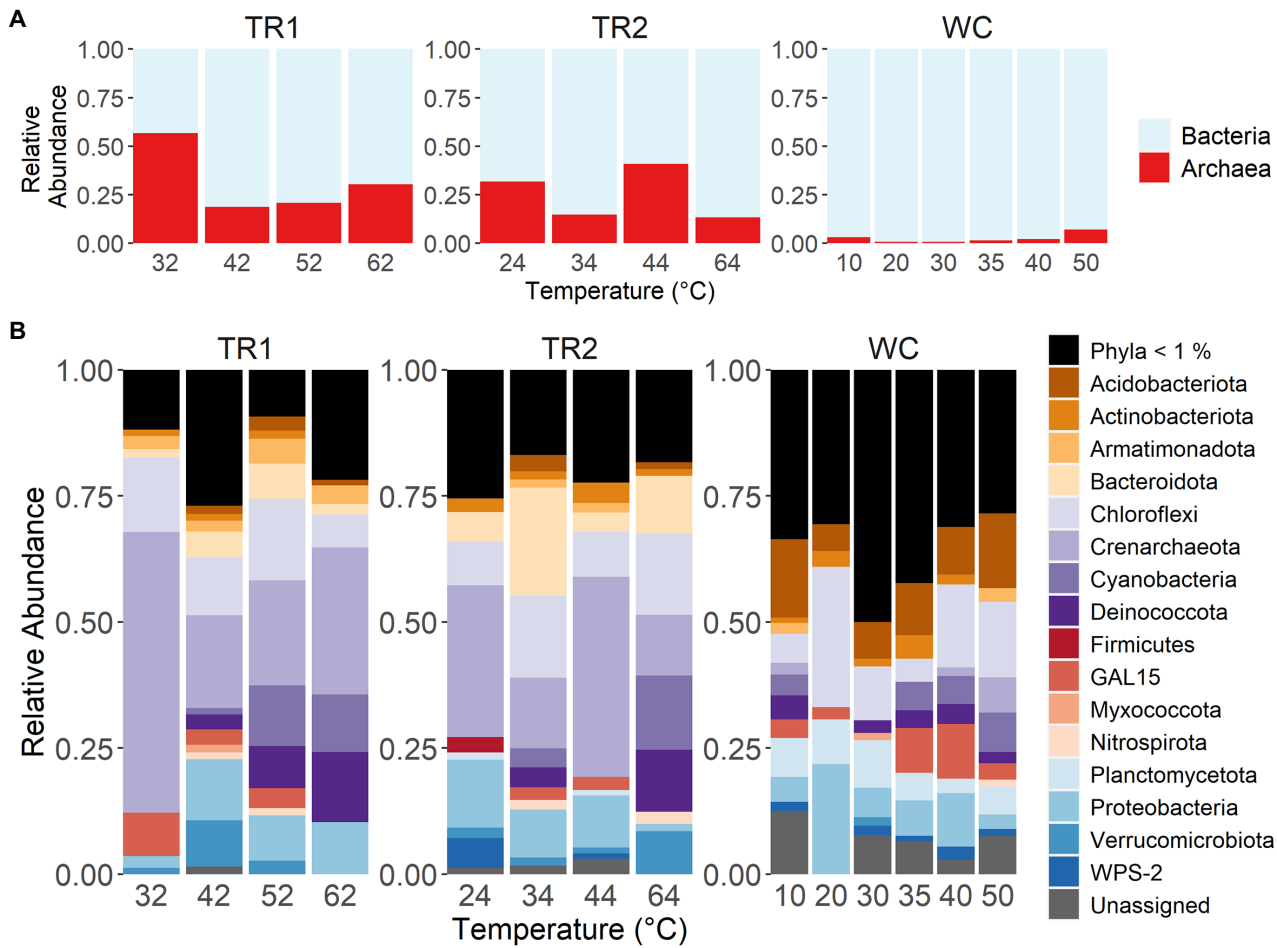
Relative abundance of either **(A)** Archaea or Bacteria and **(B)** most abundant phyla (> 1%) at the three transects studied. Samples are ordered on the x-axis in order of increasing temperature within transect. TR1: Tramway Ridge transect 1; TR2: Tramway Ridge transect 2; WC: Western Crater transect.

We initially noticed several domain and phylum-level differences in the microbial communities between the two sites. At the domain level, Tramway Ridge samples showed much higher relative sequence abundances (13%–57%) of Archaea than Western Crater samples (0.5%–7%; Figure 4A). The greatest relative abundance of Archaea was found in sample TR1-32, where ASV 1 (phylum Crenarchaeota) dominated the sample set (57% of reads; Figure 4B). Notably, there were many more ASVs from low abundance phyla (< 1% relative sequence abundance) at Western Crater, which contributes to explaining the greater alpha diversity observed there (Supplementary Figure 5B). Proteobacteria and Chloroflexi were the only phyla present across all samples (Figure 4B).

**Figure 5:**
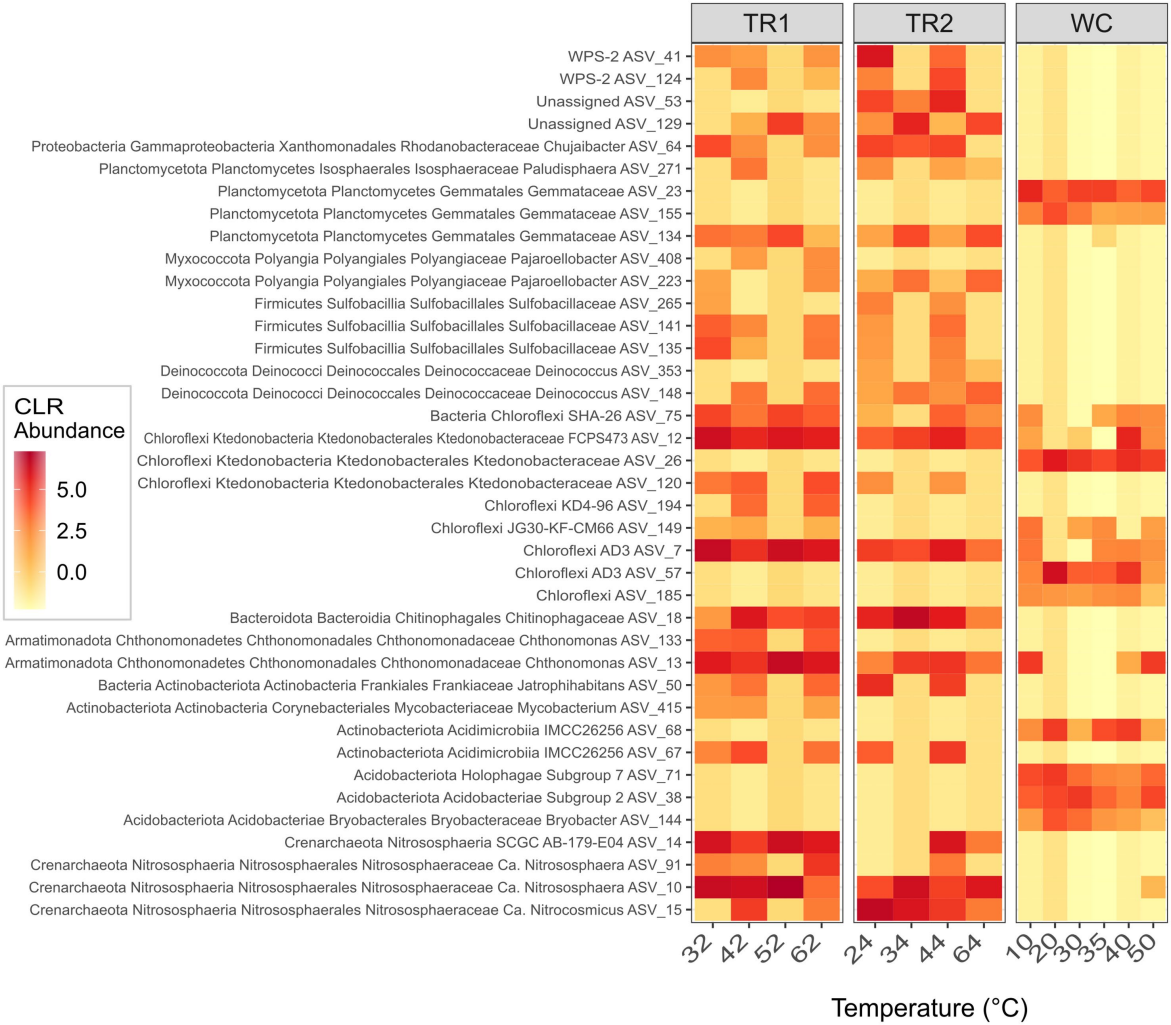
Center log-ratio transformed (CLR) abundance data for the ASVs identified by the Random Forest algorithm as being most important (mean decrease in Gini value, MDG, > 0.06) for discriminating between the microbial communities at the three transects. ASVs are ordered by domain, then phylum, in ascending order. The lowest assignable taxonomic assignments are also given. The bottom four ASVs are Archaea, the rest are Bacteria. On the x-axis, samples are ordered by increasing temperature within each transect. TR1: Tramway Ridge transect 1; TR2: Tramway Ridge transect 2; WC: Western Crater transect; The full taxonomy of these ASVs is given in Supplementary Table 4.

To identify the key groups of ASVs that reflected differences in the microbial communities between the two sites and within Tramway Ridge transects, a Random Forest classification algorithm was used. The algorithm successfully discriminated between samples from Western Crater and Tramway Ridge (area under curve, AUC, 1, *p* = 0.02) as well as samples from all three transects (AUC 1, *p* = 0.003). Center log-ratio transformed (CLR) sequence abundances for the ASVs that were found to be the most important for discriminating between the three transects (mean decrease in Gini value, MDG, > 0.06) are shown in Figure 5, with full taxonomy given in Supplementary Table 4.

The abundance profiles of ASVs from several phyla were important for the algorithm to distinguish between the communities at the two sites. ASVs from a few phyla (primarily Acidobacteriota, Chloroflexi, and Planctomycetota) were present at Western Crater, not Tramway Ridge (Figure 5, Supplementary Table 4). On the other hand, ASVs from multiple phyla were present at Tramway Ridge, not Western Crater; of note are ASVs from Crenarchaeota, Chloroflexi, Deinococcota, and Firmicutes. Several ASVs that were primarily found at Tramway Ridge were constrained to one of the two Tramway Ridge transects, allowing discrimination between transects. ASVs primarily found in TR1 samples and not TR2 include ASVs from several phyla, especially Actinobacteriota and Chloroflexi (Figure 5, Supplementary Table 4). Only a few ASVs from a variety of phyla (including unassigned) were primarily found in TR2 samples, but not TR1 (Figure 5, Supplementary Table 4).

When ASVs from this study were aligned against the NCBI 16S rRNA gene sequence database, five were found to have 100% identity to sequences found at Tramway Ridge in previous studies (Soo et al., 2009; Herbold et al., 2014a; Supplementary Table 3). Of these, two (ASV 5 and 24) were from Deinococcota, genus *Meiothermus* (Supplementary Table 3). The others were ASV 36 from Cyanobacteria, genus *Fischerella* PCC-9339, ASV 61 from Acidobacteriota, genus *Pyrinomonas*, and ASV 47 from Chloroflexi, genus *Anaerolinae* (Supplementary Table 3). ASV 61 was the only ASV detected at both sites, the others being exclusive to Tramway Ridge.

### Network Analysis and Modularity

**Figure 6:**
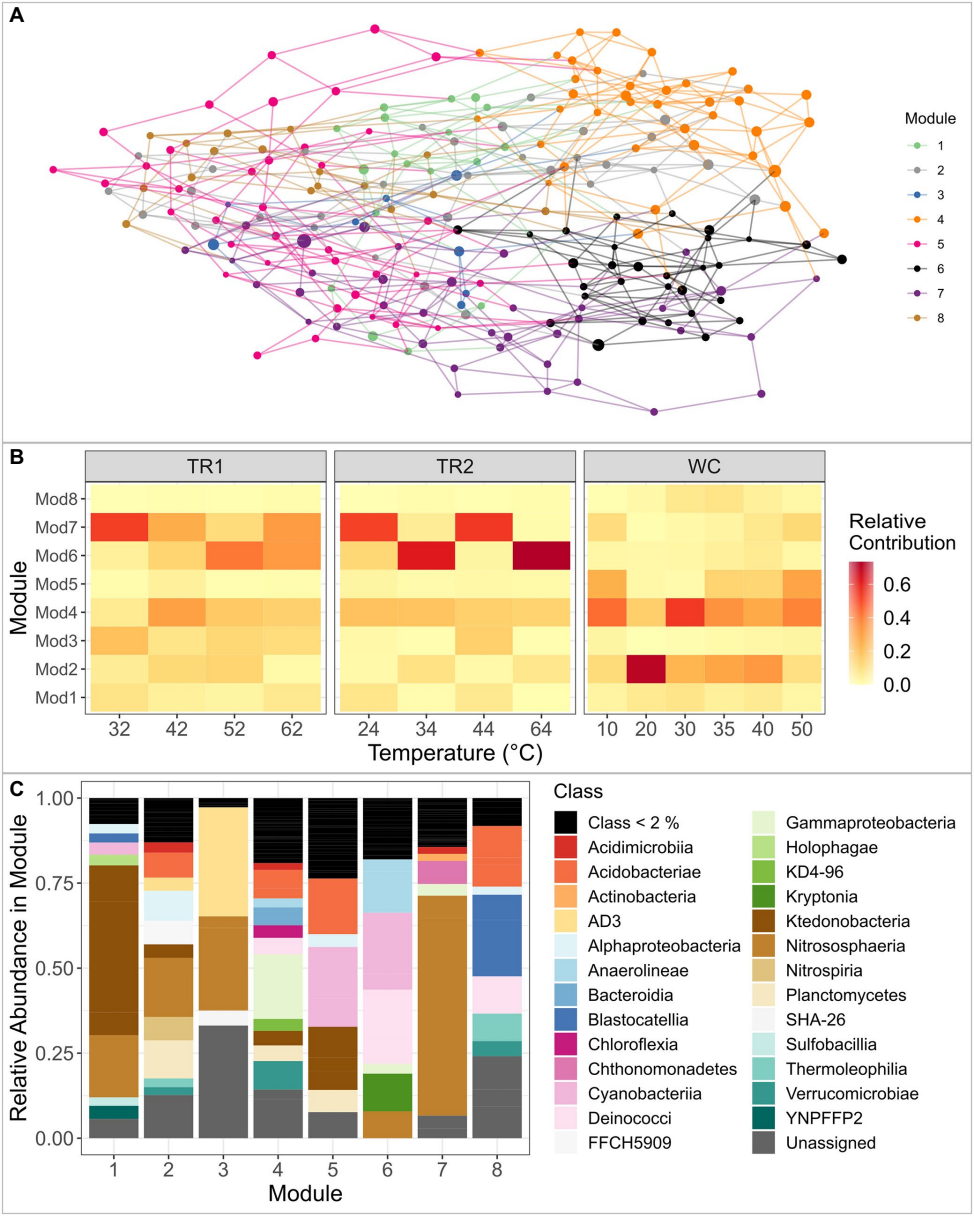
**(A)** Visual representation of the undirected co-variation network of the trimmed ASV data set from all samples. Nodes and lines are color-coded by module, as defined by the igraph fast greedy modularity optimization algorithm. Node size reflects the mean center log-ratio abundance of the ASV across all samples. **(B)** Heat map of the relative contribution of ASVs from each of the modules to the total microbial community in each sample, clustered by transect. The x-axis is ordered by increasing sample temperature within transect. TR1: Tramway Ridge 1 transect; TR2: Tramway Ridge 2 transect; WC: Western Crater transect. **(C)** Microbial composition of each module from the network analysis ordered alphabetically. Size of the bars reflects the relative abundance of that class within the module. Only classes with a relative abundance above 2% are plotted for ease of visualization.

To better understand ASV co-occurrence and identify sets of ASVs that are potentially adapted to similar environmental conditions, we constructed a network of the abundant ASVs in our data set (trimmed to retain ASVs with >10 reads in >3 samples, 217 ASVs; Figure 6). The algorithm divided the network into eight distinct modules that contain ASVs with similar abundance and frequency profiles (Figure 6A). The network had a modularity of 0.58, indicating a relatively high connection of nodes within modules. The relative contribution of each module in each sample can be found in Figure 6B and the taxonomic composition of each module is displayed in Figure 6C, with full taxonomy in Supplementary Table 5.

Modules 2 and 4 were found across almost all samples and were very diverse in taxonomic composition; the greatest abundance classes were Nitrososphaeria (phylum Crenarchaeota) and Gammaproteobacteria for Modules 2 and 4, respectively (Figures 6B,C, Supplementary Table 5). Module 7 was comprised primarily of Nitrososphaeria (58% of reads in module) and was mainly present at Tramway Ridge (Figure 6B,C, Supplementary Table 5). Module 6 was similarly more abundant at Tramway Ridge but was at high abundance in samples where Module 7 was at low abundance within Tramway Ridge 2, and vice versa (Figure 6B). The predominant members of Module 6 were from classes Cyanobacteriia (family Leptolyngbyaceae), Deinococci, and Anaerolineae (phylum Chloroflexi; Figure 6C, Supplementary Table 5). Module 3 was mainly present at Tramway Ridge, especially TR1 (Figure 6B), and was comprised of ASVs from the phylum GAL15 and classes Nitrososphaeria and AD3 (phylum Chloroflexi; Figure 6C, Supplementary Table 5). Modules 5 and 8 were constrained mostly to high-temperature samples from Western Crater; Module 5 had a majority of ASVs from classes Acidobacteriotae (phylum Acidobacteriota), Ktedonobacteria (phylum Chloroflexi), and Cyanobacteriia (family Nostocaceae), while Module 8 had a similar preponderance of ASVs from phylum Acidobacteriota, in addition to ASVs from class Blastocatellia (Figures 6B,C).

## Discussion

16S rRNA gene amplicon sequencing was used to examine the microbial community structure and composition at two disparate geothermal sites on Mt. Erebus, Tramway Ridge, and Western Crater, as well as at two distinct transects within the highly heterogeneous Tramway Ridge site. The pairing of the biological data with physicochemistry and elemental analyses allowed for insights into the impact of temperature and geochemistry on the soil microbial communities. Our key findings were as: the two sites had pronounced differences in soil physicochemistry and microbial communities, with pH having the strongest correlation with microbial communities; differential abundance profiles of several key ASVs from a few phyla were primarily responsible for the different microbial communities; and differences in physicochemistry and microbial communities were not as distinct within Tramway Ridge.

### Abiotic Drivers of Microbial Communities Between Sites

Despite Tramway Ridge and Western Crater being only 1 km apart on the summit of Mt. Erebus, they have distinct physicochemical characteristics. This is apparent from the site-specific clustering of samples in the PCoA, the significant differences between the two sites in measured soil parameters and concentrations of multiple elements, the differences between sites in how soil physicochemistry varied down the temperature gradient (Figure 2, Supplementary Figure 2). Our measured values across all parameters were within the ranges of previously reported values from Tramway Ridge (Soo et al., 2009; Herbold et al., 2014a; Vickers et al., 2016) and Western Crater (Hudson and Daniel, 1988).

We found that pH, not temperature, had the strongest correlation with community composition (Figure 3A). pH has previously been found to be a major driver of microbial community structure both at geothermal sites and in non-geothermal soils, especially below 70°C (Fierer, 2017; Power et al., 2018). Other studies at geothermal sites have found soil temperature to be a strong determinant of microbial community composition (Cole et al., 2013; Wang et al., 2013; Sharp et al., 2014; Guo et al., 2020). A variety of other factors were correlated with the microbial community composition of the two sites, including total nitrogen (TN), as was observed at a geothermal site on Deception Island, Antarctica (Bendia et al., 2018).

The differences in soil physicochemistry between the two sites despite their proximity can be traced to differences in geothermal features (e.g., active fumaroles at Tramway Ridge but not Western Crater) and exposure level to extreme weather events. First, the greater exposure to weather and lack of fumarolic steam at Western Crater likely leads to drier soil than at the relatively more sheltered Tramway Ridge, resulting in the difference in soil moisture (GWC) observed (Figure 2A). The alkaline soil pH measured at Western Crater, which is similar to pH measurements at other sites on the Mt. Erebus summit (Hudson and Daniel, 1988), reflects the alkaline nature of the phonolite lava flows dominating the summit of Mt. Erebus (Kelly et al., 2008; Panter and Winter, 2008). On the other hand, the low pH of Tramway Ridge is somewhat anomalous for the Mt. Erebus summit; several factors may be driving this lower pH at Tramway Ridge, which are likely primarily geological in nature with possible minor contributions by putative S-oxidizing taxa. One significant contributing factor may be the elevated levels of S at Tramway Ridge (Figure 2C), which *via* a combination of abiotic and biotic inputs (e.g., minor levels of sulfur-oxidizing *Sulfobacillaceae* ASVs–Supplementary Table 5), may contribute to the acidification of Tramway Ridge soils (Soo et al., 2009; Sims et al., 2020). These higher S levels, in turn, may result from a shallower heat and gas source at Tramway Ridge than Western Crater, as evidenced by the higher surface temperatures at Tramway Ridge, resulting in less opportunities for the gaseous S [mostly SO_2_ and COS (Radke, 1982)] to be filtered out by the bedrock. Another factor could be increased levels of CO_2_ at Tramway Ridge due to the presence of active fumaroles, which could produce increased levels of carbonic acid in the soil (Andrews and Schlesinger, 2001).

The more protected nature of Tramway Ridge has allowed for the establishment of extensive moss beds and cyanobacterial mats near the hot fumaroles (Broady, 1984; Soo et al., 2009; Bolhuis et al., 2014), which in turn contribute to the significantly elevated total carbon (TC), TN, and DNA yields measured (Figure 2A, Supplementary Table 1). In particular, the elevated TN levels present may be driven by the diazotrophic activity of Cyanobacteria and symbiotic microbes living in the mosses; at least one known diazotrophic Cyanobacteria was detected at Tramway Ridge in our analysis, Leptolyngbyaceae (Yoon et al., 2017). The higher TN levels at Tramway Ridge could also be influenced by the lower pH present, which would trap ammonia gases as ammonium (Holloway et al., 2011). Previous studies have suggested that moss beds and cyanobacterial mats increase the abundance and diversity of bacteria and other infauna relative to surrounding soils, which could account for the higher DNA yields observed at Tramway Ridge (Carreira et al., 2015; Prieto-Barajas et al., 2018).

### Biological Differences Between Sites

The microbial communities at Tramway Ridge and Western Crater were significantly different, as supported by the separate clustering of samples from the two sites in the dbRDA, a statistically significant PERMANOVA test, and the ability of the Random Forest algorithm to distinguish between the communities at the two sites (Figures 3A, 5). Geothermal features often contain spatially close sites that harbor unique microbial communities due to steep differences in chemical parameters (Takacs-Vesbach et al., 2008; Power et al., 2018). Differential abundance profiles for ASVs from a variety of phyla drive these differences in community composition.

We found a higher abundance of ASVs from Nitrososphaerales (phylum Crenarchaeota, also known as Thaumarchaeota in some papers (Brochier-Armanet et al., 2008), including past studies at Mt. Erebus (Soo et al., 2009; Herbold et al., 2014a) and known as thermoproteota in the genome taxonomy database (GTDB; Rinke et al., 2021)) and Nitrospira (phylum Nitrospirae) at Tramway Ridge compared to Western Crater (Figures 4, 6). This could potentially indicate increased nitrogen cycling at Tramway Ridge compared to Western Crater, since most Nitrososphaerales exhibit an ammonia-oxidizing phenotype [ammonia-oxidizing Archaea (AOA)] (Pester et al., 2011) and Nitrospira are their nitrite-oxidizing bacteria (NOB) counterparts (Gu et al., 2017; Parada and Fuhrman, 2017). Some potential sources for the ammonia used by the AOA could be nitrogen fixation performed by diazotrophic Cyanobacteria present or the trapping of ammonium due to low pH, as discussed above. These AOA and NOB may be absent from Western Crater due to the lower TN levels at Western Crater compared to Tramway Ridge (Figure 2A). Members of Nitrososphaeria have been found in previous studies at geothermal sites, especially fumarole-associated sites and within fumarole steam (Benson et al., 2011; Cole et al., 2013; Cockell et al., 2019), as well as at Tramway Ridge (Soo et al., 2009; Herbold et al., 2014a).

Results from our co-variation network analysis, which groups ASVs into distinct modules based on similar abundance profiles, provided additional insight into why Nitrososphaerales were more abundant at Tramway Ridge. ASVs from Nitrososphaerales co-occurred with ASVs from the phylum GAL15 and the class AD3 (now Candidate phylum Dormibacteraeota (Ji et al., 2017)) in Module 3 and with ASVs from class Chthonomonadetes (Lee et al., 2011) in Module 7 (Figure 6). The dominance of these taxa within these modules likely reflects the ecosystems they experience on Mt. Erebus; GAL15 and AD3 taxa have been found to inhabit relatively oligotrophic, subsurface soils at non-geothermal sites with high moisture (Frey et al., 2016, 2021; Brewer et al., 2019), and members of Chthonomonadetes primarily grow in moderately acidic geothermal soils and are oligotrophic carbohydrate-utilizing heterotrophs (Stott et al., 2008). The co-occurrence of many Nitrososphaerales ASVs with these groups points to a mutual requirement for the types of low pH and high GWC soils found at Tramway Ridge, as previously shown for AOA in the Dry Valleys of Antarctica (Magalhães et al., 2014).

Differences in abundance profiles of several bacterial groups can similarly be traced to differences in metabolic strategy and growth requirements between groups. The higher soil pH and lower EC at Western Crater resulted in ASVs from Acidobacteriota being found only at Western Crater (Figures 4–6). Acidobacteriota are generally found at alkaline, low EC soils in non-geothermal areas (Dunbar et al., 1999; Ward et al., 2009; Feeser et al., 2018). ASVs from Firmicutes, order Sulfobacillales, were more abundant at Tramway Ridge (Figures 4–6) due to their predicted reliance on sulfur oxidation for energy and the higher levels of sulfur found at Tramway Ridge (Figure 2C; Justice et al., 2014). Members of Bacteroidota and Deinococcus (genus *Meiothermus*) are known to mainly degrade complex organic compounds, which are often produced by phototrophs (Thomas et al., 2011; Albuquerque et al., 2018). Their abundance at Tramway Ridge could be linked to the phototrophic biomass present, as indicated by their co-occurrence with Cyanobacteria (in Module 6; Figure 6C) and as previously observed at Tramway Ridge (Herbold et al., 2014a) and other sites in Antarctica (Stanish et al., 2013; Coyne et al., 2020). However, we note that due to the constraints of our sample set (small sample size and samples from very different sites; Berry and Widder, 2014), our co-occurrence network cannot predict whether there are interactions occurring between these degraders of complex organics and the Cyanobacteria present in the same Module.

### Differences in Physicochemistry and Biology Within Tramway Ridge

Although the differences in geochemistry and microbial communities within Tramway Ridge are not as pronounced as those between Tramway Ridge and Western Crater, our observations highlight the large heterogeneity within this site. The ratio of Pb isotopes in soil can be used to provide information on the geological source rock composition (Doe et al., 1966), as previously done at Mt. Erebus (Sims et al., 2008; Gili et al., 2016). The marked difference we observed in average Pb^208^/Pb^207^ ratios between the two Tramway Ridge transects (Supplementary Figure 3) indicate that these two, close-proximity transects differ in initial U/Th/Pb ratios of the source rock, implying different mineralogical compositions at sites within Tramway Ridge that could influence the microbial community (Mitchell et al., 2013). Differences in soil pH and elemental concentrations between the two transects (Supplementary Figures 4A,C), combined with the broad range of pH (3.1–8.1) measured there previously (Hudson and Daniel, 1988; Soo et al., 2009; Herbold et al., 2014a; Vickers et al., 2016), suggest that different fumaroles at Tramway Ridge produce different surface chemistries, as hypothesized previously (Ilanko et al., 2019). These different chemistries, in turn, drive different microbial communities. A clear instance of this is the high abundance of *Leptolyngbyaceae* ASVs and associated heterotrophs (Module 6) at the two high pH samples within Tramway Ridge 2, correlated with the high Cu, Fe, and Zn present (Figure 6, Supplementary Table 5).

A previous study at Tramway Ridge also found a significant effect of pH and TC on community composition between samples taken close to fumaroles vs. those further from fumaroles (Soo et al., 2009). In contrast to previous studies of Tramway Ridge that only found low (~1%) relative abundance of Proteobacteria at a few sample sites (Soo et al., 2009; Herbold et al., 2014a), we found Proteobacteria across all Tramway Ridge samples at relative abundances above 5% (Figure 4). This could be due to differences in the 16S rRNA gene primers used between our study and previous studies, since previous 16S rRNA gene primers have been found to underestimate Alphaproteobacteria abundance (Apprill et al., 2015; Parada et al., 2016). On the other hand, we did not find large numbers of Planctomycetota in any of our Tramway Ridge samples (<2% relative abundance), despite their abundance in previous studies (15% in an early paper and 4% in a later paper that corroborated 16S amplicon sequences with full metagenomic 16S gene sequences; Soo et al., 2009; Herbold et al., 2014a). The primer set we used in our study is known to capture Planctomycetota abundance better than the previous set (Parada et al., 2016), so primer bias cannot explain the lack of Planctomycetota in our samples. The difference could instead be due to the high heterogeneity present at Tramway Ridge, which is also a potential explanation for the observed difference in Proteobacteria abundance.

### Potential for Phototrophy and Chemolithotrophy to Support Microbial Communities

We speculate that the majority of the microbial communities at Tramway Ridge and Western Crater is supported either *via* phototrophy, based on the presence of Cyanobacteria and associated heterotrophic degraders of complex organic molecules, or chemolithotrophy. We detected many ASVs that are most closely related to microbes with capacities for several chemolithotrophic metabolisms; based on ASV abundance (Figures 4, 6B,C, Supplementary Tables 4, 5), we predict that the most abundant chemolithotrophic processes occurring in our samples are nitrogen metabolism, gas metabolism, and Fe metabolism. For nitrogen metabolism, these metabolic capacities were for ammonia oxidation (Nitrososphaerales), nitrite oxidation (Nitrospira and *Nitrolancea*), or nitrate reduction (*Mizugakiibacter*; Brochier-Armanet et al., 2012; Kojima et al., 2014; Sorokin et al., 2014). For gas metabolism, these metabolic capacities were for H_2_ oxidation, CO oxidation, and autotrophic CO_2_ fixation. A wide range of ASVs found in our samples are most closely related to microbes with capacities for metabolism of these gases; these include ASVs from Chloroflexi classes Ktedonobacteria, Chloroflexia, and AD3 (Candidate phylum Dormibacteraeota), as well as ASVs from WPS-2 [candidate phylum Eremiobacteriota (Ji et al., 2017)], *Candidatus* Udaeobacter, and *C.* Koribacter (Ward et al., 2009; Ji et al., 2017; Brewer et al., 2019; Islam et al., 2019; Willms et al., 2020). For Fe metabolism, we detected multiple *Acidibacter* ASVs (Fe reduction; Falagán and Johnson, 2014) and Acidimicrobiia ASVs (Fe oxidation; Clark and Norris, 1996). All of these metabolites (TN, H_2_, CO, CO_2_, and Fe) were either detected in our samples (TN and Fe; Figure 2) or have been measured at these sites before (H_2_, CO, and CO_2_; Oppenheimer and Kyle, 2008; Ilanko et al., 2019)_._ All of these metabolic predictions based on 16S rRNA gene sequence similarity will need to be further tested using metagenomic and culture-based experiments.

## Conclusion

This research has examined microbial communities from two disparate geothermal sites on Mt. Erebus, finding unique microbial communities at these two sites correlated with physicochemical differences, especially soil pH. Previous microbial community studies of Mt. Erebus have focused on Tramway Ridge, but the unique and rich microbial communities found at Western Crater point to the importance of further studies at sites across Mt. Erebus to better understand this exceptional habitat. Cultivation of microbial representatives combined with in-depth studies of metagenomes from these sites are needed to test the predictions about microbial metabolic functions generated from our study and gain greater insight into the potentially endemic microorganisms inhabiting Mt. Erebus.

## Data Availability Statement

The datasets presented in this study can be found in online repositories. The names of the repository/repositories and accession number(s) can be found at: https://www.ncbi.nlm.nih.gov/, PRJNA760951.

## Author Contributions

SC, IM, CL, JA, and MS designed the study and provided funding. IM, CL, and MS collected the samples. ES did the DNA extractions and sample processing for ICP-MS. MB and ES performed initial DNA sequence processing and analysis. SN and MB completed the analyses and wrote the manuscript based on the thesis written by ES, with input from SC. Later versions were based on input and suggestions from all authors. All authors contributed to the article and approved the submitted version.

## Funding

This work was supported by the Royal Society of New Zealand (Marsden Grant 18-UOW-028 to SC, MS, IM, CL, and JA).

## Conflict of Interest

The authors declare that the research was conducted in the absence of any commercial or financial relationships that could be construed as a potential conflict of interest.

## Publisher’s Note

All claims expressed in this article are solely those of the authors and do not necessarily represent those of their affiliated organizations, or those of the publisher, the editors and the reviewers. Any product that may be evaluated in this article, or claim that may be made by its manufacturer, is not guaranteed or endorsed by the publisher.

## Footnotes

^1^ https://doi.org/10.5281/zenodo.3986799

^2^ https://www.inkscape.org
