# Supplementary material for: Unique Geothermal Chemistry Shapes Microbial Communities on Mt. Erebus, Antarctica
Source: Front Microbiol. 2022 May 3;13:836943. doi: 10.3389/fmicb.2022.836943 (PMC9111169; doi:10.3389/fmicb.2022.836943)
Supplement: Supplementary file 1 [file Data_Sheet_1.PDF]

Supplementary Material

1 Supplementary Figures and Tables

1.1 Supplementary Tables

Supplementary Table 1. Soil physicochemistry measurements and amount of DNA extracted (grams of soil, wet weight) across all samples.

	**Tramway Ridge 1 (TR1)**				**Tramway Ridge 2 (TR2)**				**Western Crater (WC)**					
**Sample**	TR1-62	TR1-52	TR1-42	TR1-32	TR2-64	TR2-44	TR2-34	TR2-24	WC-50	WC-40	WC-35	WC-30	WC-20	WC-10
**Temperature (°C)**	62	52	42	32	64	44	34	24	50	40	35	30	20	10
**pH**	4.41	4.74	4.75	4.04	6.2	4.72	5.75	4.53	9.2	8.88	8.08	7.62	7.43	6.93
**Conductivity (µS/cm)**	228.4	455	216.4	183.4	313	218	957	290	6.39	4.26	4.89	5.09	5.63	6.39
**Total Carbon, TC (%)**	1.015	2.078	1.123	0.638	2.904	0.338	1.294	0.355	1.156	0.178	0.287	0.115	0.165	0.082
**Total Nitrogen, TN (%)**	0.253	0.461	0.367	0.132	0.404	0.057	0.305	0.149	0.206	0.037	0.052	0.026	0.037	0.023
**TC:TN**	4.01	4.51	3.06	4.83	7.19	5.93	4.24	2.38	5.61	4.81	5.52	4.42	4.46	3.57
**Gravitational Water Content, GWC (%)**	44.82	63.52	39.02	29.08	41.64	43.19	42.33	49.83	23.03	24.26	26.42	19.21	35.88	30.13
**DNA (µg DNA/g soil)**	1.87	2.68	2.66	2.52	3.30	2.91	3.34	0.02	0.053	0.009	0.146	0.164	0.005	0.049

Supplementary Table 2. Levels of all elements (ordered by increasing atomic weight) analysed via ICP-MS, with values given in parts per billion (ppb). ND: not detected. Rows highlighted are those referred to in the main text.

	**Tramway Ridge 1**				**Tramway Ridge 2**				**Western Crater**					
Sample Temp. (°C)	*62*	*52*	*42*	*32*	*64*	*44*	*34*	*24*	*50*	*40*	*35*	*30*	*20*	*10*
**^{11}B**	76.503	81.826	78.143	81.665	82.114	82.945	84.522	79.89	81.289	78.971	81.408	81.269	79.717	80.877
**^{23}Na**	3338.1	8281.3	4047.4	9613	21196	8422.7	12883	2693.6	14741	7357.3	10993	11190	10571	10365
**^{24}Mg**	2258.7	2722.4	2243	3176.8	4201.9	5531.5	2435.4	2299.3	3482.4	6449.2	5445.7	5214.1	7565.6	5277.5
**^{27}Al**	117670	112740	142520	46659	76003	84470	93913	39088	79960	73970	83806	59022	87389	79692
**^{31}P**	1730.2	1513	1552.6	1815.5	1283.9	2538.7	1227.5	873.16	1249.8	2535.1	2252.2	2270.5	2783.8	2646.5
**^{32}S**	968.65	865.78	597.41	956.1	866.02	264.4	799.18	1598.2	1781.1	137.89	290.49	248.89	219.32	245.43
**^{39}K**	2435.7	5979.5	2692.6	6519	14835	6283.7	9131	2109.4	10331	5703.6	8383.9	7430.7	8032.5	7864.5
**^{40}Ca**	2557.9	5109.5	5053.7	4375.8	6711.5	7687.3	3322.2	1569	4535.8	8037	7907.7	7018.9	8597	7752.4
**^{51}V**	1.294	2.413	1.73	2.724	8.546	0.759	4.738	7.285	6.659	1.246	ND	ND	2.455	0.836
**^{52}Cr**	50.252	39.113	17.223	44.199	58.341	41.99	50.47	23.373	29.254	57.738	64.816	75.629	87.779	59.748
**^{55}Mn**	1809.2	2177.7	1366.5	1663.9	1732.1	1174.7	2024.4	2540.6	1816.3	1208	1373.9	1041.1	1370	1106.7
**^{56}Fe**	24367	17128	10924	39936	69909	17816	57662	61122	75346	19035	17977	17489	21114	20185
**^{59}Co**	1.243	1.428	1.058	1.94	2.585	1.489	2.044	2.402	2.44	1.618	1.486	1.625	2.174	1.855
**^{59}Ni**	0.655	0.552	0.478	2.844	3	0.75	1.626	0.96	1.707	0.928	1.596	1.16	1.417	1.802
**^{64}Cu**	1.093	0.941	0.487	1.781	2.255	0.916	2.259	1.646	3.087	0.809	0.986	1.131	1.296	1.111
**^{65}Zn**	55.024	54.026	36.329	74.481	121.96	48.533	114	85.937	121.98	45.918	46.432	52.473	65.978	58.086
**^{68}Zn**	66.828	69.679	49.149	79.655	123.98	58.073	114.75	90.237	121.82	54.086	55.681	57.924	72.283	63.533
**^{75}As**	11.455	14.091	16.553	8.309	11.163	9.674	5.071	5.35	7.416	10.983	10.044	6.944	8.565	8.351
**^{79}Se**	10.566	11.225	13.879	2.995	3.838	8.152	2.45	2.393	2.774	9.128	8.188	5.893	7.336	6.824
**^{82}Se**	19.94	20.369	25.213	5.03	6.199	14.874	4.508	4.832	4.497	15.803	15.171	10.141	12.512	11.625
**^{88}Sr**	23.825	32.352	27.305	50.597	99.537	50.286	62.984	44.925	68.456	59.711	52.737	47.565	52.86	47.927
**^{91}As**	1.41	4.05	3.64	6.24	9.132	2.122	3.139	4.236	5.481	2.661	1.926	1.599	1.72	1.793
**^{112}Cd**	2.501	5.17	4.529	0.771	2.544	0.423	4.22	0.948	0.716	0.419	0.467	0.326	0.382	0.264
**^{137}Ba**	943.81	1237.1	1161.2	298.78	374.69	637.62	283.63	295.18	257.92	589.81	640.35	353.47	507.75	388.23
**^{204}Tl**	0.17	0.273	0.238	0.106	0.123	0.091	0.202	0.169	0.08	0.072	0.099	0.074	0.065	0.045

**^{206}Pb**	33.273	32.603	26.884	30.97	55.925	11.55	15.377	15.403	40.238	10.677	12.116	7.46	8.898	7.335
**^{207}Pb**	29.736	29.116	23.971	27.691	49.926	10.344	13.743	13.697	35.97	9.579	10.791	6.668	7.977	6.582
**^{208}Pb**	32.49	31.642	26.1	30.023	54.064	10.855	14.431	14.385	39.11	10.024	11.342	6.999	8.356	6.889
**^{238}U**	12.119	11.502	14.628	5.152	8.782	11.735	6.611	5.485	7.464	11.193	11.616	8.062	10.216	9.127

Supplementary Table 3. Accession numbers for the five ASVs from this study that showed 100% similarity to 16S rRNA gene amplicon sequences from previous Mt. Erebus studies.

ASV #	SILVA classification	Best hit from NCBI Nucleotide Collection	% Similarity	NCBI Accession # for top hit	Reference for top hit
ASV_5	Bacteria, Deinococcota, Deinococci, Thermales, Thermaceae, Meiothermus	Uncultured bacterium clone ERBTW20082009	100	KF923322.1	(Herbold et al., 2014)
ASV_24	Bacteria, Deinococcota, Deinococci, Thermales, Thermaceae, Meiothermus	Uncultured bacterium clone ERBTW20082009	100	KF923322.1	(Herbold et al., 2014)
ASV_36	Bacteria, Cyanobacteria, Cyanobacteriia, Cyanobacteriales, Nostocaceae, Fischerella PCC-9339	Uncultured bacterium clone ERBTW20082009OTU014	100	KF923320.1	(Herbold et al., 2014)
ASV_47	Bacteria, Chloroflexi, Anaerolineae, SBR1031, NA, NA	Uncultured bacterium clone ERB-B6	100	EU490269.1	(Soo et al., 2009)
ASV_61	Bacteria, Acidobacteriota, Blastocatellia, Pyrinomonadales, Pyrinomonadaceae, Pyrinomonas	Uncultured bacterium clone ERBTW20082009OTU078	100	KF923318.1	(Herbold et al., 2014)

Supplementary Table 4. SILVA-based taxonomy of the ASVs identified as most important for distinguishing the microbial communities at the three transects, in the same order as they appear in the heatmap (Figure 6). Mean decrease in Gini value (MDG) is a measure of the importance of that ASV for distinguishing the three transects. No species designations could be made for any ASV.

ASV	MDG	Domain	Phylum	Class	Order	Family	Genus
ASV_41	0.08318	Bacteria	WPS-2	Unclassified	Unclassified	Unclassified	Unclassified
ASV_124	0.060728	Bacteria	WPS-2	Unclassified	Unclassified	Unclassified	Unclassified
ASV_53	0.070816	Bacteria	Unclassified	Unclassified	Unclassified	Unclassified	Unclassified
ASV_129	0.065076	Bacteria	Unclassified	Unclassified	Unclassified	Unclassified	Unclassified
ASV_64	0.062706	Bacteria	Proteobacteria	Gammaproteobacteria	Xanthomonadales	Rhodanobacteraceae	Chujaibacter
ASV_134	0.067998	Bacteria	Planctomycetota	Planctomycetes	Gemmatales	Gemmataceae	Unclassified
ASV_155	0.063898	Bacteria	Planctomycetota	Planctomycetes	Gemmatales	Gemmataceae	Unclassified
ASV_23	0.062073	Bacteria	Planctomycetota	Planctomycetes	Gemmatales	Gemmataceae	Unclassified
ASV_271	0.061492	Bacteria	Planctomycetota	Planctomycetes	Isosphaerales	Isosphaeraceae	Paludisphaera
ASV_408	0.072961	Bacteria	Myxococcota	Polyangia	Polyangiales	Polyangiaceae	Pajaroellobacter
ASV_223	0.066339	Bacteria	Myxococcota	Polyangia	Polyangiales	Polyangiaceae	Pajaroellobacter
ASV_141	0.078646	Bacteria	Firmicutes	Sulfobacillia	Sulfobacillales	Sulfobacillaceae	Unclassified
ASV_135	0.069741	Bacteria	Firmicutes	Sulfobacillia	Sulfobacillales	Sulfobacillaceae	Unclassified
ASV_265	0.069638	Bacteria	Firmicutes	Sulfobacillia	Sulfobacillales	Sulfobacillaceae	Unclassified
ASV_353	0.0865	Bacteria	Deinococcota	Deinococci	Deinococcales	Deinococcaceae	Deinococcus
ASV_148	0.069879	Bacteria	Deinococcota	Deinococci	Deinococcales	Deinococcaceae	Deinococcus
ASV_7	0.092013	Bacteria	Chloroflexi	AD3	Unclassified	Unclassified	Unclassified
ASV_149	0.091037	Bacteria	Chloroflexi	JG30-KF-CM66	Unclassified	Unclassified	Unclassified
ASV_12	0.083285	Bacteria	Chloroflexi	Ktedonobacteria	Ktedonobacterales	Ktedonobacteraceae	FCPS473
ASV_120	0.071097	Bacteria	Chloroflexi	Ktedonobacteria	Ktedonobacterales	Ktedonobacteraceae	Unclassified
ASV_194	0.069304	Bacteria	Chloroflexi	KD4-96	Unclassified	Unclassified	Unclassified
ASV_26	0.06881	Bacteria	Chloroflexi	Ktedonobacteria	Ktedonobacterales	Ktedonobacteraceae	Unclassified
ASV_57	0.065705	Bacteria	Chloroflexi	AD3	Unclassified	Unclassified	Unclassified
ASV_75	0.062799	Bacteria	Chloroflexi	SHA-26	Unclassified	Unclassified	Unclassified
ASV_185	0.061564	Bacteria	Chloroflexi	Unclassified	Unclassified	Unclassified	Unclassified
ASV_18	0.062354	Bacteria	Bacteroidota	Bacteroidia	Chitinophagales	Chitinophagaceae	Unclassified

ASV_133	0.12304	Bacteria	Armatimonadota	Chthonomonadetes	Chthonomonadales	Chthonomonadaceae	Chthonomonas
ASV_13	0.122575	Bacteria	Armatimonadota	Chthonomonadetes	Chthonomonadales	Chthonomonadaceae	Chthonomonas
ASV_415	0.13942	Bacteria	Actinobacteriota	Actinobacteria	Corynebacteriales	Mycobacteriaceae	Mycobacterium
ASV_50	0.07261	Bacteria	Actinobacteriota	Actinobacteria	Frankiales	Frankiaceae	Jatrophihabitans
ASV_67	0.069084	Bacteria	Actinobacteriota	Acidimicrobiia	IMCC26256	Unclassified	Unclassified
ASV_68	0.065406	Bacteria	Actinobacteriota	Acidimicrobiia	IMCC26256	Unclassified	Unclassified
ASV_38	0.068488	Bacteria	Acidobacteriota	Acidobacteriae	Subgroup 2	Unclassified	Unclassified
ASV_144	0.061618	Bacteria	Acidobacteriota	Acidobacteriae	Bryobacterales	Bryobacteraceae	Bryobacter
ASV_71	0.06079	Bacteria	Acidobacteriota	Holophagae	Subgroup 7	Unclassified	Unclassified
ASV_15	0.133396	Archaea	Crenarchaeota	Nitrososphaeria	Nitrososphaerales	Nitrososphaeraceae	Candidatus Nitrosocosmicus
ASV_14	0.114836	Archaea	Crenarchaeota	Nitrososphaeria	SCGC AB-179-E04	Unclassified	Unclassified
ASV_10	0.097116	Archaea	Crenarchaeota	Nitrososphaeria	Nitrososphaerales	Nitrososphaeraceae	Candidatus Nitrososphaera
ASV_91	0.076107	Archaea	Crenarchaeota	Nitrososphaeria	Nitrososphaerales	Nitrososphaeraceae	Candidatus Nitrososphaera

Supplementary Table 5. SILVA-based taxonomy of module members from the network analysis. Frequency given is calculated based on the abundance of that ASV within the module. No species assignments could be made for any ASVs.

Mod	ASV	Freq	Domain	Phylum	Class	Order	Family	Genus
1	ASV_12	0.3246	Bacteria	Chloroflexi	Ktedonobacteria	Ktedonobacterales	Ktedonobacteraceae	FCPS473
1	ASV_27	0.1833	Archaea	Crenarchaeota	Nitrososphaeria	Nitrososphaerales	Nitrososphaeraceae	Candidatus Nitrososphaera
1	ASV_46	0.1072	Bacteria	Chloroflexi	Ktedonobacteria	Ktedonobacterales	Ktedonobacteraceae	FCPS473
1	ASV_63	0.067	Bacteria	Chloroflexi	Ktedonobacteria	Ktedonobacterales	Ktedonobacteraceae	G12-WMSP1
1	ASV_72	0.0565	Bacteria	Unclassified	Unclassified	Unclassified	Unclassified	Unclassified
1	ASV_102	0.0387	Bacteria	Firmicutes	YNPFFP2	Unclassified	Unclassified	Unclassified
1	ASV_106	0.0358	Bacteria	Cyanobacteria	Cyanobacteriia	Leptolyngbyales	Leptolyngbyaceae	Leptolyngbya PCC-6306
1	ASV_116	0.0313	Bacteria	Acidobacteriota	Holophagae	Subgroup 7	Unclassified	Unclassified
1	ASV_125	0.0277	Bacteria	Proteobacteria	Alpha-proteobacteria	Rhizobiales	Xanthobacteraceae	Unclassified
1	ASV_128	0.027	Bacteria	Acidobacteriota	Blastocatellia	Blastocatellales	Blastocatellaceae	Unclassified
1	ASV_135	0.0246	Bacteria	Firmicutes	Sulfobacillia	Sulfobacillales	Sulfobacillaceae	Unclassified
1	ASV_177	0.0152	Bacteria	Proteobacteria	Alpha-proteobacteria	Rhizobiales	Xanthobacteraceae	Unclassified
1	ASV_199	0.0129	Bacteria	Planctomycetota	Planctomycetes	Gemmatales	Gemmataceae	Unclassified
1	ASV_217	0.0114	Bacteria	Chloroflexi	Anaerolineae	SBR1031	Unclassified	Unclassified
1	ASV_218	0.0113	Bacteria	Planctomycetota	Planctomycetes	Gemmatales	Gemmataceae	Unclassified
1	ASV_236	0.01	Bacteria	Unclassified	Unclassified	Unclassified	Unclassified	Unclassified
1	ASV_237	0.0099	Bacteria	Actinobacteriota	Acidimicrobiia	IMCC26256	Unclassified	Unclassified
1	ASV_328	0.0055	Bacteria	Myxococcota	Polyangia	Haliangiales	Haliangiaceae	Haliangium
2	ASV_10	0.174	Archaea	Crenarchaeota	Nitrososphaeria	Nitrososphaerales	Nitrososphaeraceae	Candidatus Nitrososphaera
2	ASV_16	0.1269	Bacteria	GAL15	Unclassified	Unclassified	Unclassified	Unclassified
2	ASV_32	0.0734	Bacteria	Acidobacteriota	Acidobacteriae	Bryobacterales	Bryobacteraceae	Bryobacter
2	ASV_33	0.0729	Bacteria	Planctomycetota	Planctomycetes	Gemmatales	Gemmataceae	Unclassified
2	ASV_34	0.0692	Bacteria	Nitrospirota	Nitrospiria	Nitrospirales	Nitrospiraceae	Nitrospira
2	ASV_35	0.0691	Bacteria	Acidobacteriota	FFCH5909	Unclassified	Unclassified	Unclassified

2	ASV_55	0.0394	Bacteria	Chloroflexi	Ktedonobacteria	Ktedonobacterales	Ktedonobacteraceae	JG30a-KF-32
2	ASV_56	0.0389	Bacteria	Planctomycetota	Planctomycetes	Gemmatales	Gemmataceae	Unclassified
2	ASV_57	0.0388	Bacteria	Chloroflexi	AD3	Unclassified	Unclassified	Unclassified
2	ASV_60	0.0371	Bacteria	Proteobacteria	Alpha-proteobacteria	Rhizobiales	Beijerinckiaceae	Methylovirgula
2	ASV_66	0.0309	Bacteria	Proteobacteria	Alpha-proteobacteria	Sphingomonadales	Sphingomonadaceae	Unclassified
2	ASV_68	0.0302	Bacteria	Actinobacteriota	Acidimicrobiia	IMCC26256	Unclassified	Unclassified
2	ASV_77	0.0256	Bacteria	Actinobacteriota	Thermoleophilia	Gaiellales	Unclassified	Unclassified
2	ASV_85	0.0231	Bacteria	Verrucomicrobiota	Verrucomicrobiae	Chthoniobacterales	Chthoniobacteraceae	Unclassified
2	ASV_93	0.0202	Bacteria	Proteobacteria	Alpha-proteobacteria	Rhizobiales	Hyphomicrobiaceae	Pedomicrobium
2	ASV_118	0.0151	Bacteria	Proteobacteria	Gamma-proteobacteria	WD260	Unclassified	Unclassified
2	ASV_139	0.0118	Bacteria	Proteobacteria	Alpha-proteobacteria	Reyranellales	Reyranellaceae	Reyranella
2	ASV_140	0.0116	Bacteria	Proteobacteria	Alpha-proteobacteria	Rhizobiales	Beijerinckiaceae	Methylobacterium-Methylorubrum
2	ASV_144	0.0108	Bacteria	Acidobacteriota	Acidobacteriae	Bryobacterales	Bryobacteraceae	Bryobacter
2	ASV_145	0.0107	Bacteria	Methylomirabilota	Methylomirabilia	Rokubacteriales	Unclassified	Unclassified
2	ASV_155	0.0096	Bacteria	Planctomycetota	Planctomycetes	Gemmatales	Gemmataceae	Unclassified
2	ASV_176	0.0075	Bacteria	Planctomycetota	Planctomycetes	Gemmatales	Gemmataceae	Unclassified
2	ASV_178	0.0075	Bacteria	Planctomycetota	Planctomycetes	Gemmatales	Gemmataceae	Unclassified
2	ASV_185	0.0072	Bacteria	Chloroflexi	Unclassified	Unclassified	Unclassified	Unclassified
2	ASV_198	0.0064	Bacteria	Planctomycetota	Planctomycetes	Gemmatales	Gemmataceae	Unclassified
2	ASV_212	0.0057	Bacteria	Proteobacteria	Alpha-proteobacteria	Rhizobiales	Xanthobacteraceae	Unclassified
2	ASV_224	0.0053	Bacteria	Planctomycetota	Planctomycetes	Gemmatales	Gemmataceae	Unclassified
2	ASV_229	0.0052	Bacteria	Myxococcota	Polyangia	Haliangiales	Haliangiaceae	Haliangium
2	ASV_242	0.0047	Bacteria	Proteobacteria	Alpha-proteobacteria	Acetobacterales	Acetobacteraceae	Unclassified
2	ASV_245	0.0045	Bacteria	Actinobacteriota	Actinobacteria	Frankiales	Frankiaceae	Jatrophihabitans

2	ASV_289	0.0034	Bacteria	Acidobacteriota	Acidobacteriae	Solibacterales	Solibacteraceae	Candidatus Solibacter
2	ASV_295	0.0033	Bacteria	Actinobacteriota	Acidimicrobiia	Unclassified	Unclassified	Unclassified
3	ASV_6	0.3311	Bacteria	GAL15	Unclassified	Unclassified	Unclassified	Unclassified
3	ASV_7	0.3204	Bacteria	Chloroflexi	AD3	Unclassified	Unclassified	Unclassified
3	ASV_14	0.2434	Archaea	Crenarchaeota	Nitrososphaeria	SCGC AB-179-E04	Unclassified	Unclassified
3	ASV_75	0.044	Bacteria	Chloroflexi	SHA-26	Unclassified	Unclassified	Unclassified
3	ASV_91	0.0336	Archaea	Crenarchaeota	Nitrososphaeria	Nitrososphaerales	Nitrososphaeraceae	Candidatus Nitrososphaera
3	ASV_163	0.0145	Bacteria	Myxococcota	Polyangia	Haliangiales	Haliangiaceae	Haliangium
3	ASV_251	0.007	Bacteria	Chloroflexi	Ktedonobacteria	Ktedonobacterales	Ktedonobacteraceae	Unclassified
3	ASV_280	0.006	Archaea	Crenarchaeota	Nitrososphaeria	Caldiarchaeales	Terrestrial Hot Spring Gp(THSCG)	Unclassified
4	ASV_2	0.1469	Bacteria	Proteobacteria	Gamma-proteobacteria	Xanthomonadales	Rhodanobacteraceae	Mizugakiibacter
4	ASV_4	0.1148	Bacteria	Unclassified	Unclassified	Unclassified	Unclassified	Unclassified
4	ASV_8	0.0838	Bacteria	Verrucomicro-biota	Verrucomicrobiae	Chthoniobacterales	Chthoniobacteraceae	Candidatus Udaeobacter
4	ASV_17	0.0553	Bacteria	Acidobacteriota	Acidobacteriae	Acidobacteriales	Koribacteraceae	Candidatus Koribacter
4	ASV_18	0.0526	Bacteria	Bacteroidota	Bacteroidia	Chitinophagales	Chitinophagaceae	Unclassified
4	ASV_21	0.0483	Bacteria	Deinococcota	Deinococci	Thermales	Thermaceae	Meiothermus
4	ASV_23	0.0458	Bacteria	Planctomycetota	Planctomycetes	Gemmatales	Gemmataceae	Unclassified
4	ASV_25	0.0432	Bacteria	Proteobacteria	Gamma-proteobacteria	Gamma-proteobacteria Incertae Sedis	Unknown Family	Acidibacter
4	ASV_26	0.0428	Bacteria	Chloroflexi	Ktedonobacteria	Ktedonobacterales	Ktedonobacteraceae	Unclassified
4	ASV_28	0.0369	Bacteria	Chloroflexi	Chloroflexia	Thermomicrobiales	Thermomicrobiaceae	Nitrolancea
4	ASV_30	0.0354	Bacteria	Chloroflexi	KD4-96	Unclassified	Unclassified	Unclassified
4	ASV_38	0.0284	Bacteria	Acidobacteriota	Acidobacteriae	Subgroup 2	Unclassified	Unclassified
4	ASV_39	0.0281	Bacteria	WPS-2	Unclassified	Unclassified	Unclassified	Unclassified
4	ASV_42	0.0263	Bacteria	Chloroflexi	Anaerolineae	SBR1031	A4b	Unclassified

4	ASV_49	0.0201	Bacteria	Actinobacteriota	Acidimicrobiia	IMCC26256	Unclassified	Unclassified
4	ASV_54	0.0185	Bacteria	Acidobacteriota	Acidobacteriae	Acidobacteriales	Acidobacteriaceae (Subgroup 1)	Granulicella
4	ASV_62	0.0148	Bacteria	Myxococcota	Polyangia	Haliangiales	Haliangiaceae	Haliangium
4	ASV_65	0.0141	Archaea	Crenarchaeota	Nitrososphaeria	Nitrososphaerales	Nitrososphaeraceae	Candidatus Nitrososphaera
4	ASV_71	0.013	Bacteria	Acidobacteriota	Holophagae	Subgroup 7	Unclassified	Unclassified
4	ASV_79	0.0111	Bacteria	Planctomycetota	Planctomycetes	Isosphaerales	Isosphaeraceae	Unclassified
4	ASV_82	0.0107	Bacteria	Planctomycetota	Planctomycetes	Gemmatales	Gemmataceae	Unclassified
4	ASV_88	0.0096	Bacteria	Chloroflexi	AD3	Unclassified	Unclassified	Unclassified
4	ASV_89	0.0095	Bacteria	Planctomycetota	Planctomycetes	Gemmatales	Gemmataceae	Unclassified
4	ASV_90	0.0092	Bacteria	Chloroflexi	Unclassified	Unclassified	Unclassified	Unclassified
4	ASV_92	0.0089	Bacteria	Chloroflexi	Ktedonobacteria	Ktedonobacterales	Ktedonobacteraceae	1921-2
4	ASV_101	0.0084	Bacteria	Planctomycetota	Planctomycetes	Gemmatales	Gemmataceae	Unclassified
4	ASV_103	0.0083	Bacteria	Actinobacteriota	Thermoleophilia	Solirubrobacterales	Solirubrobacteraceae	Conexibacter
4	ASV_104	0.0082	Bacteria	Acidobacteriota	Acidobacteriae	Bryobacterales	Bryobacteraceae	Bryobacter
4	ASV_105	0.0082	Bacteria	Unclassified	Unclassified	Unclassified	Unclassified	Unclassified
4	ASV_109	0.0077	Bacteria	Acidobacteriota	Acidobacteriae	Solibacterales	Solibacteraceae	Candidatus Solibacter
4	ASV_113	0.0072	Bacteria	Unclassified	Unclassified	Unclassified	Unclassified	Unclassified
4	ASV_147	0.0046	Bacteria	Firmicutes	Unclassified	Unclassified	Unclassified	Unclassified
4	ASV_158	0.0041	Bacteria	Acidobacteriota	Acidobacteriae	Subgroup 2	Unclassified	Unclassified
4	ASV_159	0.0041	Bacteria	Acidobacteriota	Acidobacteriae	Bryobacterales	Bryobacteraceae	Bryobacter
4	ASV_172	0.0035	Bacteria	Acidobacteriota	Acidobacteriae	Acidobacteriales	Acidobacteriaceae (Subgroup 1)	Acidipila
4	ASV_175	0.0034	Bacteria	Planctomycetota	Pla4 lineage	Unclassified	Unclassified	Unclassified
4	ASV_191	0.003	Bacteria	Planctomycetota	Pla4 lineage	Unclassified	Unclassified	Unclassified
4	ASV_331	0.0012	Bacteria	Myxococcota	Polyangia	Haliangiales	Haliangiaceae	Haliangium
5	ASV_20	0.1636	Bacteria	Acidobacteriota	Acidobacteriae	Subgroup 2	Unclassified	Unclassified
5	ASV_40	0.0911	Bacteria	Chloroflexi	Ktedonobacteria	Ktedonobacterales	Ktedonobacteraceae	FCPS473
5	ASV_43	0.0826	Bacteria	Cyanobacteria	Cyanobacteriia	Cyanobacteriales	Nostocaceae	Mastigocladopsis PCC-10914

5	ASV_44	0.0822	Bacteria	Cyanobacteria	Cyanobacteriia	Cyanobacteriales	Nostocaceae	Unclassified
5	ASV_45	0.0765	Bacteria	GAL15	Unclassified	Unclassified	Unclassified	Unclassified
5	ASV_48	0.0698	Bacteria	Cyanobacteria	Cyanobacteriia	Cyanobacteriales	Nostocaceae	Unclassified
5	ASV_58	0.0551	Bacteria	Chloroflexi	Ktedonobacteria	Ktedonobacterales	Ktedonobacteraceae	Unclassified
5	ASV_73	0.0397	Bacteria	Chloroflexi	Ktedonobacteria	Ktedonobacterales	Ktedonobacteraceae	1921-2
5	ASV_76	0.0376	Bacteria	Proteobacteria	Alpha-proteobacteria	Rhizobiales	Xanthobacteraceae	Bradyrhizobium
5	ASV_78	0.0367	Bacteria	Planctomycetota	Planctomycetes	Gemmatales	Gemmataceae	Unclassified
5	ASV_95	0.0285	Bacteria	Planctomycetota	Planctomycetes	Gemmatales	Gemmataceae	Unclassified
5	ASV_126	0.0191	Bacteria	Unclassified	Unclassified	Unclassified	Unclassified	Unclassified
5	ASV_131	0.018	Bacteria	Cyanobacteria	Cyanobacteriia	Cyanobacteriales	Nostocaceae	Nostoc PCC-73102
5	ASV_133	0.0176	Bacteria	Armatimonadota	Chthonomonadetes	Chthonomonadales	Chthonomonadaceae	Chthonomonas
5	ASV_137	0.017	Bacteria	Gemma-timonadota	Gemmatimonadetes	Gemma-timonadales	Gemma-timonadaceae	Unclassified
5	ASV_153	0.0141	Bacteria	Myxococcota	Myxococcia	Myxococcales	Myxococcaceae	P3OB-42
5	ASV_154	0.0139	Bacteria	Proteobacteria	Alpha-proteobacteria	Rhizobiales	Xanthobacteraceae	Unclassified
5	ASV_162	0.0127	Bacteria	Proteobacteria	Alpha-proteobacteria	Caulobacterales	Caulobacteraceae	Unclassified
5	ASV_181	0.0105	Bacteria	Myxococcota	Polyangia	Polyangiales	Polyangiaceae	Pajaroellobacter
5	ASV_182	0.0104	Bacteria	Unclassified	Unclassified	Unclassified	Unclassified	Unclassified
5	ASV_184	0.0103	Bacteria	Planctomycetota	Planctomycetes	Isosphaerales	Isosphaeraceae	Unclassified
5	ASV_187	0.0102	Bacteria	Planctomycetota	Planctomycetes	Gemmatales	Gemmataceae	Unclassified
5	ASV_186	0.0102	Bacteria	Acidobacteriota	Acidobacteriae	Acidobacteriales	Acidobacteriaceae (Subgroup 1)	Acidipila
5	ASV_234	0.0071	Bacteria	Myxococcota	Myxococcia	Myxococcales	Anaero-myxobacteraceae	Anaeromyxobacter
5	ASV_239	0.0069	Bacteria	Dadabacteria	Dadabacteriia	Dadabacteriales	Unclassified	Unclassified
5	ASV_241	0.0067	Bacteria	GAL15	Unclassified	Unclassified	Unclassified	Unclassified
5	ASV_260	0.0059	Bacteria	Dependentiae	Babeliae	Babeliales	Vermiphilaceae	Unclassified
5	ASV_264	0.0058	Bacteria	Chloroflexi	Anaerolineae	SBR1031	Unclassified	Unclassified
5	ASV_265	0.0057	Bacteria	Firmicutes	Sulfobacillia	Sulfobacillales	Sulfobacillaceae	Unclassified
5	ASV_275	0.0053	Bacteria	Armatimonadota	Unclassified	Unclassified	Unclassified	Unclassified

5	ASV_284	0.0051	Bacteria	Cyanobacteria	Vampirivibrionia	Obscuribacterales	Obscuribacteraceae	Unclassified
5	ASV_291	0.0049	Bacteria	Firmicutes	Sulfobacillia	Sulfobacillales	Sulfobacillaceae	Unclassified
5	ASV_292	0.0049	Bacteria	RCP2-54	Unclassified	Unclassified	Unclassified	Unclassified
5	ASV_311	0.0044	Bacteria	WPS-2	Unclassified	Unclassified	Unclassified	Unclassified
5	ASV_318	0.0042	Bacteria	Chloroflexi	Ktedonobacteria	B10-SB3A	Unclassified	Unclassified
5	ASV_345	0.0034	Bacteria	Proteobacteria	Alpha-proteobacteria	Rhizobiales	Xanthobacteraceae	Unclassified
5	ASV_415	0.0022	Bacteria	Actinobacteriota	Actinobacteria	Corynebacteriales	Mycobacteriaceae	Mycobacterium
6	ASV_3	0.1802	Bacteria	Cyanobacteria	Cyanobacteriia	Leptolyngbyales	Leptolyngbyaceae	Leptolyngbya PCC-6306
6	ASV_5	0.1516	Bacteria	Deinococcota	Deinococci	Thermales	Thermaceae	Meiothermus
6	ASV_9	0.1219	Bacteria	Chloroflexi	Anaerolineae	Unclassified	Unclassified	Unclassified
6	ASV_11	0.1107	Bacteria	Bacteroidota	Kryptonia	Kryptoniales	BSV26	Unclassified
6	ASV_19	0.079	Archaea	Crenarchaeota	Nitrososphaeria	Nitrososphaerales	Nitrososphaeraceae	Candidatus Nitrosocosmicus
6	ASV_24	0.0664	Bacteria	Deinococcota	Deinococci	Thermales	Thermaceae	Meiothermus
6	ASV_36	0.0462	Bacteria	Cyanobacteria	Cyanobacteriia	Cyanobacteriales	Nostocaceae	Fischerella PCC-9339
6	ASV_47	0.0348	Bacteria	Chloroflexi	Anaerolineae	SBR1031	Unclassified	Unclassified
6	ASV_52	0.0287	Bacteria	Proteobacteria	Gamma-proteobacteria	Xanthomonadales	Rhodanobacteraceae	Mizugakiibacter
6	ASV_70	0.0197	Bacteria	Bacteroidota	Bacteroidia	Chitinophagales	Chitinophagaceae	Terrimonas
6	ASV_69	0.0197	Bacteria	Actinobacteriota	Thermoleophilia	Gaiellales	Unclassified	Unclassified
6	ASV_83	0.0161	Bacteria	Proteobacteria	Alpha-proteobacteria	Sphingomonadales	Sphingomonadaceae	Novosphingobium
6	ASV_96	0.013	Bacteria	Acidobacteriota	Blastocatellia	Blastocatellales	Blastocatellaceae	Unclassified
6	ASV_100	0.0127	Bacteria	Bacteroidota	Bacteroidia	Chitinophagales	Chitinophagaceae	Flaviaesturariibacter
6	ASV_115	0.0106	Bacteria	Bacteroidota	Kryptonia	Kryptoniales	BSV26	Unclassified
6	ASV_121	0.0095	Bacteria	Bacteroidota	Bacteroidia	Chitinophagales	Chitinophagaceae	Unclassified
6	ASV_122	0.0095	Bacteria	Actinobacteriota	Acidimicrobiia	Unclassified	Unclassified	Unclassified
6	ASV_129	0.0086	Bacteria	Unclassified	Unclassified	Unclassified	Unclassified	Unclassified
6	ASV_134	0.0082	Bacteria	Planctomycetota	Planctomycetes	Gemmatales	Gemmataceae	Unclassified

6	ASV_136	0.008	Bacteria	Nitrospirota	Nitrospiria	Nitrospirales	Nitrospiraceae	Nitrospira
6	ASV_150	0.0068	Bacteria	Armatimonadota	Fimbriimonadia	Fimbriimonadales	Unclassified	Unclassified
6	ASV_149	0.0068	Bacteria	Chloroflexi	JG30-KF-CM66	Unclassified	Unclassified	Unclassified
6	ASV_156	0.0063	Bacteria	Armatimonadota	Chthonomonadetes	Chthonomonadales	Unclassified	Unclassified
6	ASV_173	0.0053	Bacteria	Proteobacteria	Alpha-proteobacteria	Rhizobiales	Xanthobacteraceae	Pseudolabrys
6	ASV_183	0.0048	Bacteria	Unclassified	Unclassified	Unclassified	Unclassified	Unclassified
6	ASV_207	0.0039	Bacteria	Planctomycetota	Planctomycetes	Gemmatales	Gemmataceae	Unclassified
6	ASV_223	0.0036	Bacteria	Myxococcota	Polyangia	Polyangiales	Polyangiaceae	Pajaroellobacter
6	ASV_230	0.0034	Bacteria	Chloroflexi	Ktedonobacteria	Ktedonobacterales	JG30-KF-AS9	Unclassified
6	ASV_290	0.0023	Bacteria	Proteobacteria	Alpha-proteobacteria	Rhizobiales	Xanthobacteraceae	Rhodopseudomonas
6	ASV_338	0.0017	Bacteria	Proteobacteria	Alpha-proteobacteria	Rhizobiales	Rhizobiaceae	Unclassified
7	ASV_1	0.58	Archaea	Crenarchaeota	Nitrososphaeria	SCGC AB-179-E04	Unclassified	Unclassified
7	ASV_13	0.068	Bacteria	Armatimonadota	Chthonomonadetes	Chthonomonadales	Chthonomonadaceae	Chthonomonas
7	ASV_15	0.0667	Archaea	Crenarchaeota	Nitrososphaeria	Nitrososphaerales	Nitrososphaeraceae	Candidatus Nitrosocosmicus
7	ASV_29	0.0379	Bacteria	Unclassified	Unclassified	Unclassified	Unclassified	Unclassified
7	ASV_31	0.0342	Bacteria	Proteobacteria	Gamma-proteobacteria	Gamma-proteobacteria Incertae Sedis	Unknown Family	Acidibacter
7	ASV_41	0.0284	Bacteria	WPS-2	Unclassified	Unclassified	Unclassified	Unclassified
7	ASV_50	0.0205	Bacteria	Actinobacteriota	Actinobacteria	Frankiales	Frankiaceae	Jatrophihabitans
7	ASV_51	0.0203	Bacteria	Actinobacteriota	Acidimicrobiia	Unclassified	Unclassified	Unclassified
7	ASV_64	0.0149	Bacteria	Proteobacteria	Gamma-proteobacteria	Xanthomonadales	Rhodanobacteraceae	Chujaibacter
7	ASV_67	0.0141	Bacteria	Actinobacteriota	Acidimicrobiia	IMCC26256	Unclassified	Unclassified
7	ASV_74	0.0126	Bacteria	Planctomycetota	Planctomycetes	Gemmatales	Gemmataceae	Unclassified
7	ASV_84	0.0107	Bacteria	Proteobacteria	Gamma-proteobacteria	Xanthomonadales	Rhodanobacteraceae	Rhodanobacter
7	ASV_87	0.0103	Bacteria	Firmicutes	Bacilli	Alicyclobacillales	Alicyclobacillaceae	Acidibacillus

7	ASV_110	0.0079	Bacteria	Chloroflexi	AD3	Unclassified	Unclassified	Unclassified
7	ASV_112	0.0075	Bacteria	Chloroflexi	Ktedonobacteria	Ktedonobacterales	JG30-KF-AS9	Unclassified
7	ASV_120	0.0066	Bacteria	Chloroflexi	Ktedonobacteria	Ktedonobacterales	Ktedonobacteraceae	Unclassified
7	ASV_124	0.0062	Bacteria	WPS-2	Unclassified	Unclassified	Unclassified	Unclassified
7	ASV_138	0.0054	Bacteria	Chloroflexi	Ktedonobacteria	Ktedonobacterales	Ktedonobacteraceae	1921-2
7	ASV_141	0.0052	Bacteria	Firmicutes	Sulfobacillia	Sulfobacillales	Sulfobacillaceae	Unclassified
7	ASV_148	0.0047	Bacteria	Deinococcota	Deinococci	Deinococcales	Deinococcaceae	Deinococcus
7	ASV_165	0.004	Bacteria	Proteobacteria	Gamma-proteobacteria	Xanthomonadales	Rhodanobacteraceae	Dokdonella
7	ASV_168	0.0038	Bacteria	WPS-2	Unclassified	Unclassified	Unclassified	Unclassified
7	ASV_188	0.0032	Bacteria	Proteobacteria	Gamma-proteobacteria	Burkholderiales	Comamonadaceae	Piscinibacter
7	ASV_193	0.0031	Bacteria	Armatimonadota	Armatimonadia	Armatimonadales	Unclassified	Unclassified
7	ASV_202	0.0028	Bacteria	Myxococcota	Polyangia	Polyangiales	Polyangiaceae	Pajaroellobacter
7	ASV_205	0.0027	Bacteria	Actinobacteriota	Thermoleophilia	Solirubrobacterales	Solirubrobacteraceae	Conexibacter
7	ASV_219	0.0025	Bacteria	Proteobacteria	Alpha-proteobacteria	Acetobacterales	Acetobacteraceae	Acidiphilium
7	ASV_258	0.0019	Bacteria	Actinobacteriota	Acidimicrobiia	Unclassified	Unclassified	Unclassified
7	ASV_263	0.0018	Bacteria	Proteobacteria	Alpha-proteobacteria	Rhizobiales	Xanthobacteraceae	Pseudolabrys
7	ASV_266	0.0018	Bacteria	Gemma-timonadota	Gemmatimonadetes	Gemmatimonadales	Gemma-timonadaceae	Unclassified
7	ASV_274	0.0017	Bacteria	Planctomycetota	Planctomycetes	Isosphaerales	Isosphaeraceae	Aquisphaera
7	ASV_271	0.0017	Bacteria	Planctomycetota	Planctomycetes	Isosphaerales	Isosphaeraceae	Paludisphaera
7	ASV_293	0.0016	Bacteria	Bacteroidota	Bacteroidia	Cytophagales	Hymenobacteraceae	Hymenobacter
7	ASV_299	0.0015	Bacteria	Proteobacteria	Alpha-proteobacteria	Acetobacterales	Acetobacteraceae	Acidicaldus
7	ASV_300	0.0015	Bacteria	Acidobacteriota	Acidobacteriae	Acidobacteriales	Acidobacteriaceae (Subgroup 1)	Unclassified
7	ASV_344	0.0011	Bacteria	Proteobacteria	Gamma-proteobacteria	Gamma-proteobacteria Incertae Sedis	Unknown Family	Acidibacter
7	ASV_366	0.001	Bacteria	Chloroflexi	Chloroflexia	Elev-1554	Unclassified	Unclassified

8	ASV_61	0.1662	Bacteria	Acidobacteriota	Blastocatellia	Pyrinomonadales	Pyrinomonadaceae	Pyrinomonas
8	ASV_86	0.1095	Bacteria	Deinococcota	Deinococci	Deinococcales	Deinococcaceae	Deinococcus
8	ASV_94	0.0974	Bacteria	Chloroflexi	Unclassified	Unclassified	Unclassified	Unclassified
8	ASV_98	0.0932	Bacteria	Acidobacteriota	Acidobacteriae	Acidobacteriales	Unclassified	Unclassified
8	ASV_99	0.0923	Bacteria	Unclassified	Unclassified	Unclassified	Unclassified	Unclassified
8	ASV_111	0.0809	Bacteria	Actinobacteriota	Thermoleophilia	Solirubrobacterales	Solirubrobacteraceae	Unclassified
8	ASV_117	0.0739	Bacteria	Acidobacteriota	Blastocatellia	Pyrinomonadales	Pyrinomonadaceae	RB41
8	ASV_127	0.0642	Bacteria	Acidobacteriota	Acidobacteriae	Subgroup 2	Unclassified	Unclassified
8	ASV_146	0.0513	Bacteria	WPS-2	Unclassified	Unclassified	Unclassified	Unclassified
8	ASV_160	0.0443	Bacteria	Verrucomicrobiota	Verrucomicrobiae	Unclassified	Unclassified	Unclassified
8	ASV_235	0.0239	Bacteria	Proteobacteria	Alpha-proteobacteria	Rhizobiales	Rhodomicrobiaceae	Rhodomicrobium
8	ASV_249	0.0209	Bacteria	Acidobacteriota	Acidobacteriae	Acidobacteriales	Acidobacteriaceae (Subgroup 1)	Granulicella
8	ASV_261	0.0199	Bacteria	Proteobacteria	Alpha-proteobacteria	Rhizobiales	Beijerinckiaceae	alphaI cluster
8	ASV_267	0.0186	Bacteria	Chloroflexi	Chloroflexia	Thermomicrobiales	JG30-KF-CM45	Unclassified
8	ASV_268	0.0186	Bacteria	Proteobacteria	Gamma-proteobacteria	Burkholderiales	Oxalobacteraceae	Actimicrobium
8	ASV_310	0.0147	Bacteria	Chloroflexi	Anaerolineae	Caldilineales	Caldilineaceae	Unclassified
8	ASV_367	0.0101	Bacteria	Proteobacteria	Gamma-proteobacteria	Steroidobacterales	Steroidobacteraceae	Unclassified

1.2 Supplemental Figures

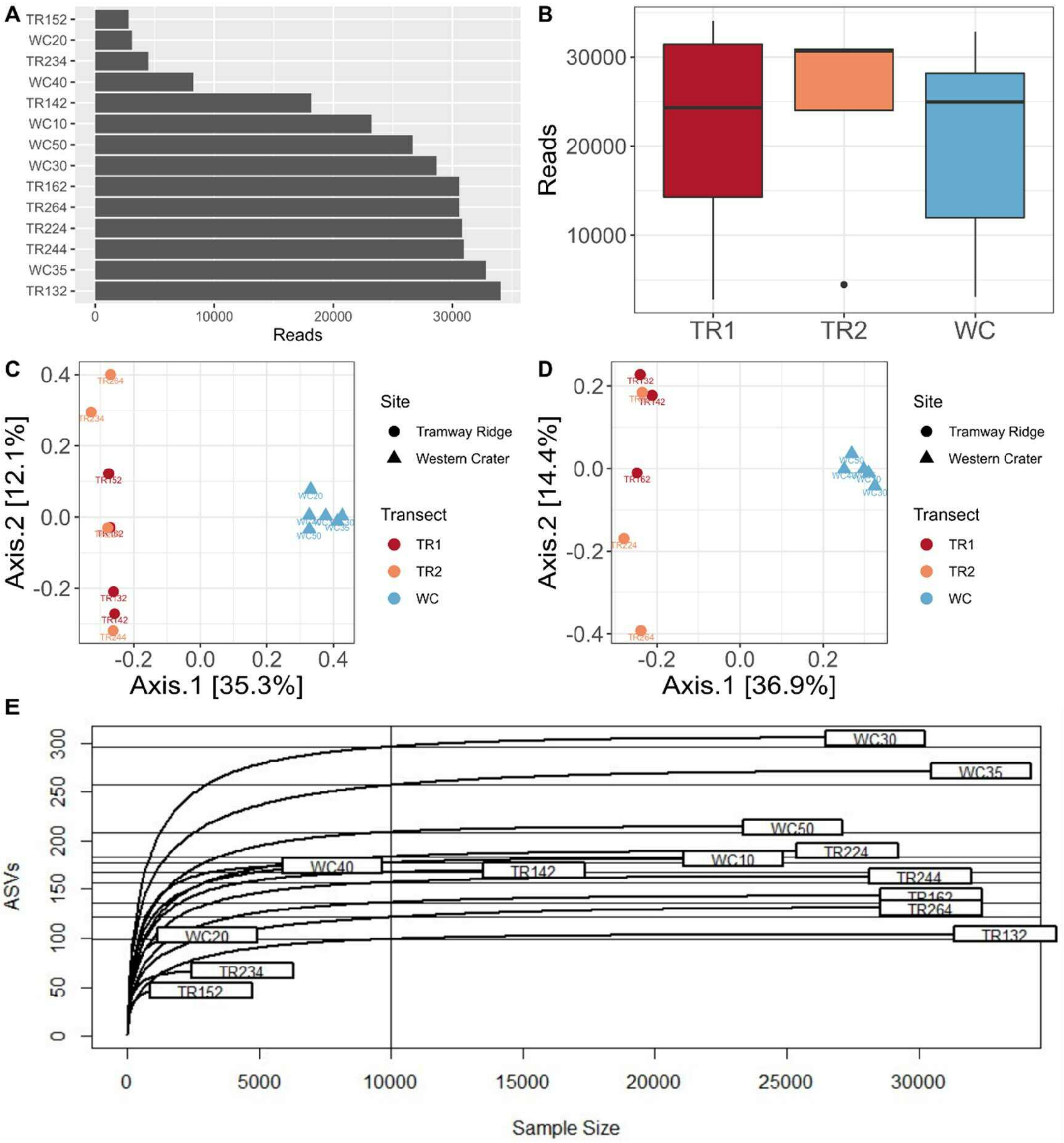

Supplementary Figure 1. (A) Read counts for 16S rRNA gene amplicon sequencing of each sample; y-axis is ordered by increasing read counts. Four samples had noticeably lower read counts than other samples. (B) Boxplot of read counts from samples, separated by transect. Median read counts from each transect were similar. (C, D) Plot of Unifrac distances between biological samples using Principal Coordinates Analysis (PCoA) either with the four low-read samples (C) or without (D). The percentage of the variation explained by the axes is given in parentheses. The variation explained is similar either with or without the four low-read samples. (E) Rarefaction curves of all biological samples. TR1: Tramway Ridge transect 1. TR2: Tramway Ridge transect 2. WC: Western Crater transect. Sample numbers correspond to the temperature of the site where that sample was collected.

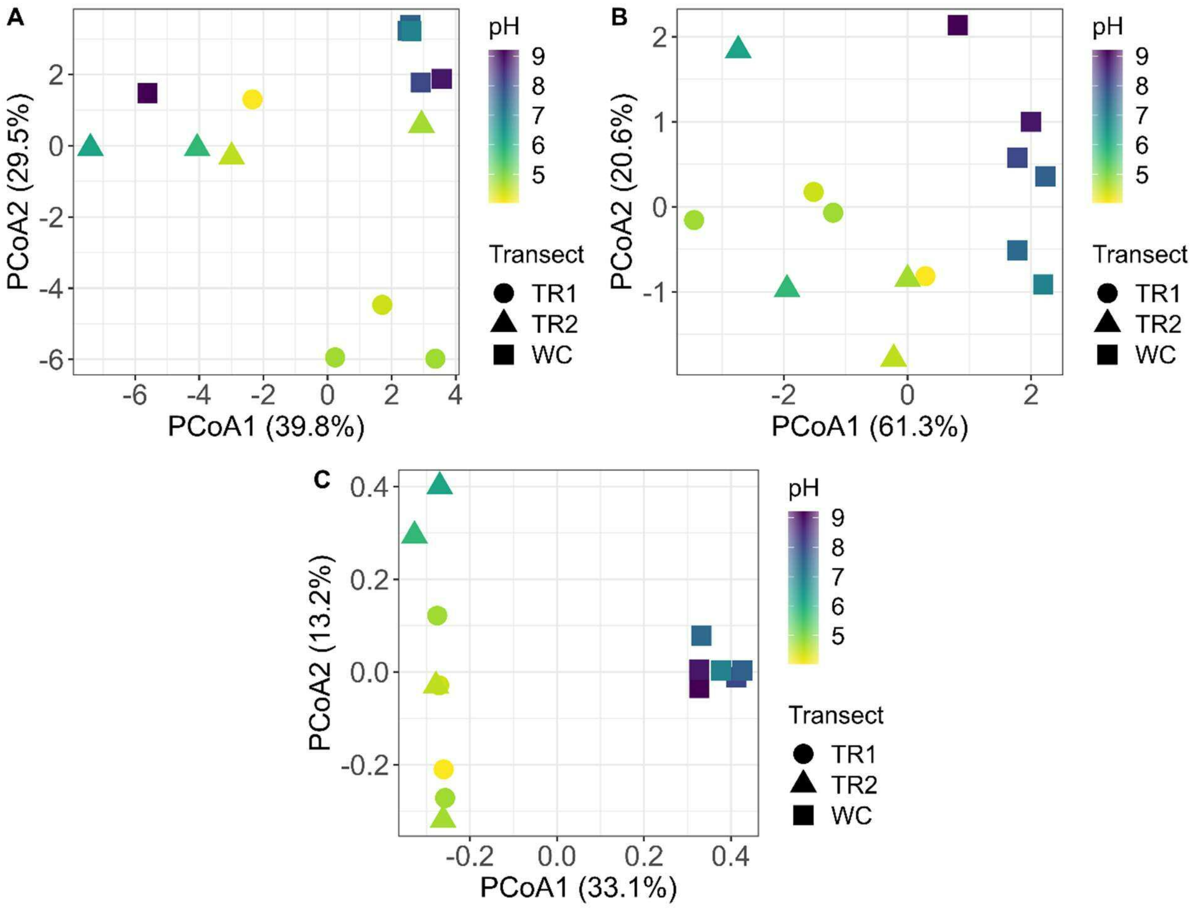

Supplementary Figure 2. (A) Principal Coordinates Analysis (PCoA) with Euclidean distances of all environmental and elemental data from all samples. (B) Same plot as in (A), but no elemental data were included; the environmental data included were pH, temperature, conductivity, C, N, C:N, and gravimetric water content. (C) PCoA plot with Unifrac distances of the microbial communities at each sample based on center log-ratio transformed abundance of 16S rRNA ASVs. Samples that are more similar to each other cluster together. The percentage given on the axes indicates the percentage of variation in the data explained by that axis. For each plot, the color of the point indicates the pH of that sample, while the shape reflects the transect of origin for that sample. TR1: Tramway Ridge transect 1. TR2: Tramway Ridge transect 2. WC: Western Crater transect.

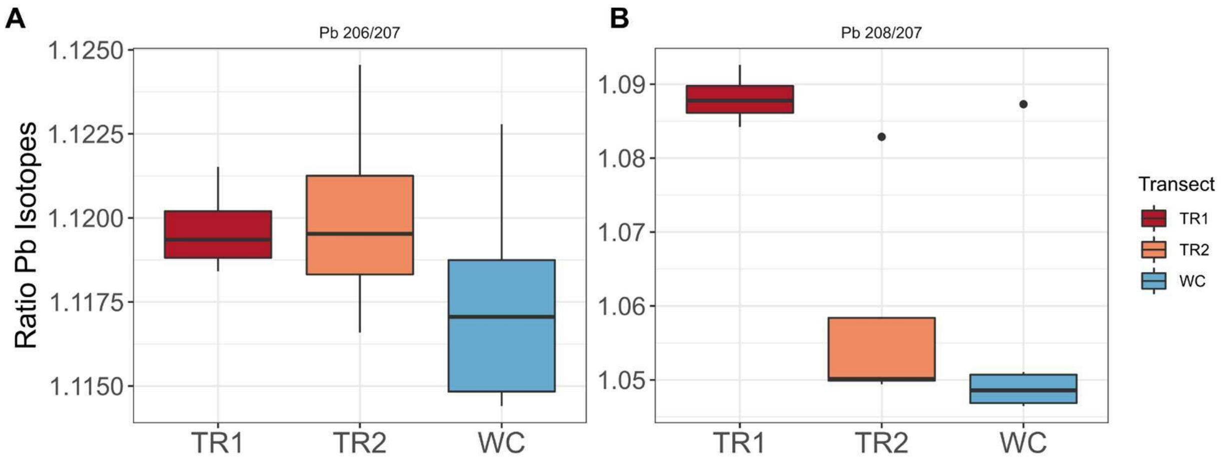

Supplementary Figure 3. Comparison of (A) Pb 206 / 207 ratios and (B) Pb 208 / 207 ratios at the three transects sampled, indicating different source rock compositions between transects. TR1: Tramway Ridge transect 1. TR2: Tramway Ridge transect 2. WC: Western Crater transect.

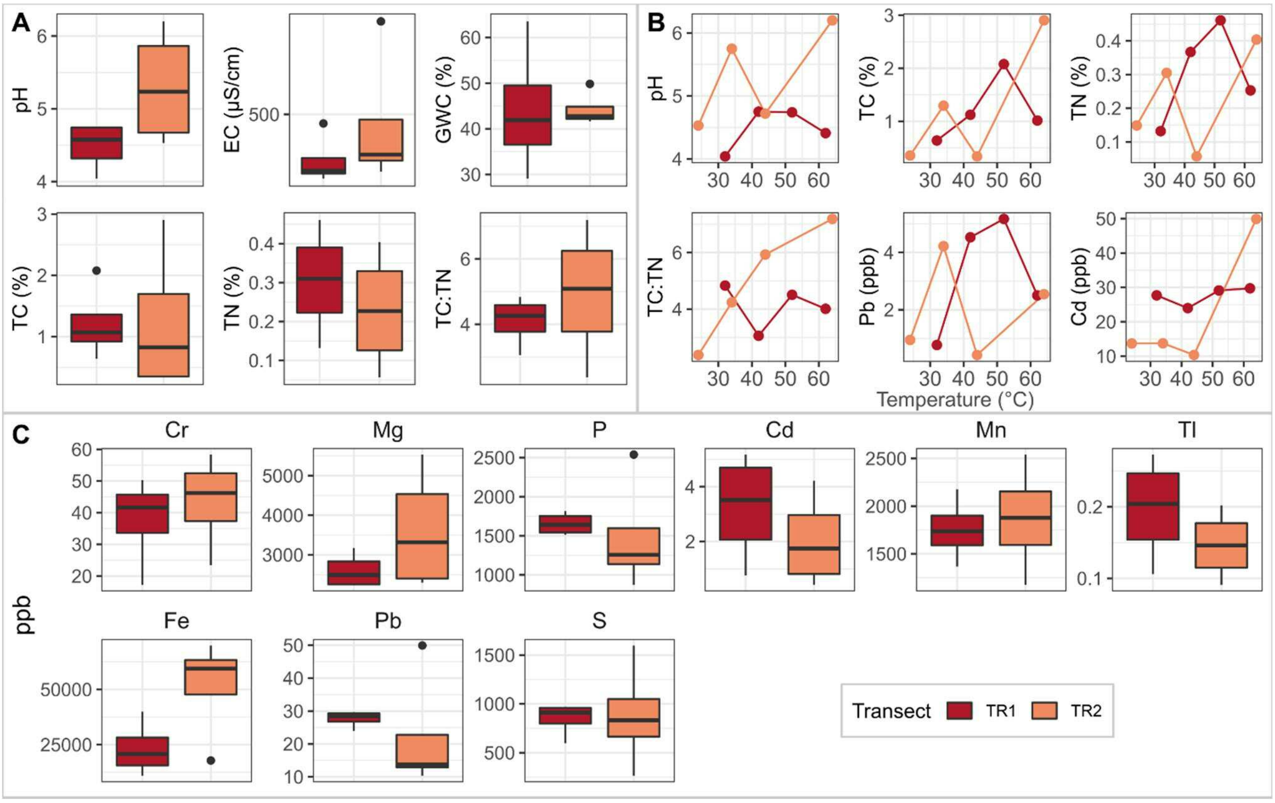

Supplementary Figure 4 Comparison of soil physicochemistry between Tramway Ridge transect 1 (TR1) and transect 2 (TR2). (A, C) Boxplots for (A) each environmental parameter and (C) elements of interest. (B) Line plots showing the variation with temperature of environmental parameters and elements of interest. Elements presented throughout this figure are the same as those in Figure 2 for ease of comparison. No significant differences were found between the two transects for any environmental parameter or element, and no significant correlations were found between temperature and any environmental parameters or elements. Ppb: concentration in parts per billion. GWC: gravitational water content. TC: total carbon. TN: total nitrogen.

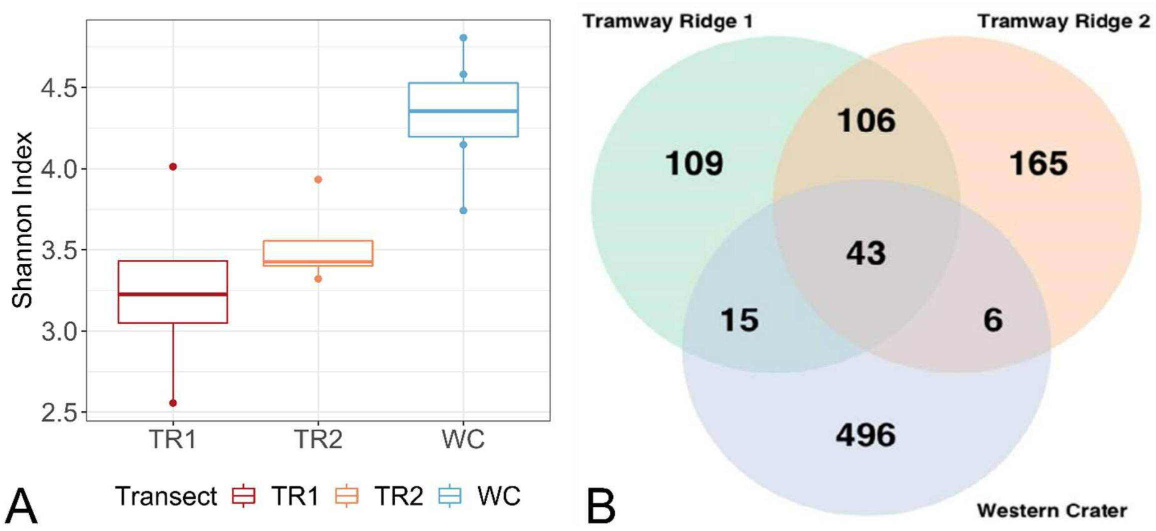

Supplementary Figure 5. (A) Plot of the α-diversity of each transect, as measured by the Shannon Index. (B) Venn diagram of ASVs either unique to each transect or shared between the transects. Total number of ASVs across all samples was 940. TR1: Tramway Ridge transect 1. TR2: Tramway Ridge transect 2. WC: Western Crater transect.
